# Acute and chronic effects of cumulative vs. continuous physical activity on cardiovascular metabolic risk biomarkers in adults: a systematic review and meta-analysis

**DOI:** 10.3389/fphys.2026.1804202

**Published:** 2026-08-28

**Authors:** Huanpu Zhang, Jiang Liu, Wenyu Zhang

**Affiliations:** Physical Culture Institute, Southwest Jiaotong University, Chengdu, Sichuan, China

**Keywords:** accumulated exercise, cardiovascular health, continuous exercise, exercise snack, fragmented exercise

## Abstract

**Objective:**

Current systematic reviews and meta-analyses comparing the effects of accumulated versus continuous exercise on cardiometabolic risk markers in adults remain scarce, and conclusions from existing primary studies are inconsistent. This study aimed to: (1) quantify the acute and chronic intervention effects of accumulated bouts of exercise versus continuous exercise on cardiometabolic risk markers in adults; (2) objectively identify between-group differences across all outcome indicators, so as to provide evidence-based references for the optimization of exercise prescription.

**Methods:**

Databases were searched from inception to January 7, 2026, with records imported into EndNote 20. Based on the PICOS framework, two researchers independently screened literature, extracted data, and resolved discrepancies via discussion or third-party arbitration. Study quality was assessed using the PEDro and MINORS scales, and bias risk via Cochrane RoB2 and ROBINS-I tools. Stata 16.0 was used for statistical analysis with a random-effects model, supplemented by sensitivity analyses and Egger’s/Begg’s tests for publication bias.

**Results:**

A total of 8,053 identified studies, 35 were included after de-duplication and full-text review. Included studies had acceptable quality and controllable bias risk; results were robust with no significant publication bias. In long-term interventions, accumulated exercise showed a small beneficial effect on weight reduction (g=-0.20, P = 0.035). For core indicators (BF, LBM, WC, blood lipids), the two modalities were equivalent. After excluding high-heterogeneity studies, their blood pressure regulatory effects were consistent. In acute interventions, accumulated exercise exerted time-dependent effects on S-SBP (advantage within 10–20 min, g=-0.61, P = 0.005) and S-DBP (reduction within 20–40 min, g=-0.61, P = 0.018), and demonstrated small-to-moderate/moderate advantages in improving S-TG-iAUC (g=0.24, P = 0.03) and S-ASI (g=-0.41, P = 0.028), with more pronounced benefits in the ΔCAVI subgroup (g=-0.56, P = 0.006).

**Conclusion:**

Compared with continuous exercise, volume-matched accumulated exercise yields statistical equivalence for most long-term cardiometabolic risk biomarkers, with only trivial statistically significant differences observed in body weight. Acute single-bout accumulated exercise merely induces transient improvements in partial blood pressure, vascular and lipid indicators without sustained long-term benefits. The evidence-based findings herein support the formulation of individualized exercise prescriptions based on participants’ time adherence and provide objective quantitative evidence to underpin accumulated exercise interventions.

## 1 Introduction

Cardiovascular Diseases (CVDs) represent a major global public health concern and are also the leading cause of death worldwide (Adhikary et al., 2022; Chomiuk et al., 2025). Data from the Global Burden of Disease Study (GBD) 2023 (Hay et al., 2025)indicated that in 2023, the number of global deaths related to CVDs reached 19.2 million, accounting for one-third of the total deaths; the cumulative number of prevalent cases stood at 626 million, doubling that of 1990. The aforementioned data highlight the severe threat posed by CVDs to the health of the global population and further exacerbate the disease burden. Therefore, exploring safe, effective, and scalable intervention strategies for the prevention and control of CVDs is of great practical significance. A large body of studies has confirmed that physical activity is a key intervention for the prevention and control of CVDs, with a well-established cardiovascular protective effect (Warburton et al., 2006; Ricci and Cunha, 2020). A single session of physical activity can rapidly reduce blood pressure levels and improve cardiometabolic risk markers such as lipid profiles (Haskell, 1994; Cardoso et al., 2010). In contrast, long-term regular physical exercise can optimize cardiovascular physiological functions from multiple dimensions, including enhancing cardiorespiratory endurance, regulating the balance of blood pressure and lipid metabolism, and improving vascular endothelial function (Haskell, 1994; Warburton et al., 2006; Cardoso et al., 2010), ultimately reducing the incidence risk of CVDs through multiple pathways.

The current consensus recommendation in clinical and public health fields is that adults should accumulate at least 150 minutes of moderate-intensity aerobic exercise per week to fully exert its core role in cardiovascular health maintenance (Kim et al., 2025). However, during practical promotion, most adults find it difficult to set aside continuous time periods for traditional continuous exercise. Therefore, shortening the duration of each exercise session may be a more promising strategy to boost exercise participation (Yin et al., 2025), and a potential alternative is accumulated exercise. Accumulated exercise refers to splitting traditional continuous exercise into multiple shorter exercise bouts, which are completed several times a day to accumulate a total exercise volume and intensity equivalent to those of continuous exercise (Murphy et al., 2009; Murphy et al., 2019; Yin et al., 2025). This exercise modality is more compatible with the fast-paced and time-fragmented lifestyle of modern adults, which may effectively improve exercise adherence and thus expand the coverage of exercise interventions (Yin et al., 2025).

Nevertheless, although the practical convenience of accumulated exercise has been widely recognized, controversies remain regarding the equivalence or differences in its effects compared with continuous exercise in improving cardiovascular health, especially in the specific manifestations of acute immediate effects and long-term chronic effects, for which a unified conclusion has not yet been reached. Further collation of previous studies reveals that comparative systematic reviews and meta-analyses on this topic are extremely scarce, and existing findings have obvious limitations, with none focusing on cardiovascular outcomes. Loh et al. (2020) focused on the comparison between interrupting sedentary behavior with intermittent exercise (INT), prolonged sedentary behavior (SIT), and single-bout continuous exercise (EX), with the core focus only on metabolic markers such as blood glucose and insulin. Murphy et al. conducted comparative studies on the two exercise modalities in 2009 (Murphy et al., 2009) and 2019 (Murphy et al., 2019), respectively; however, due to the limited number of included studies, they only preliminarily explored indicators such as blood pressure and blood lipids without in-depth verification, nor did they investigate the acute effects of exercise and cardiovascular outcomes. The study by Zhang et al. (2022) was limited to the effects of the two exercise modes on postprandial blood glucose, insulin, and triglyceride (TG). Even though Yin et al. (2025) attempted to conduct a comprehensive analysis by integrating the above research findings, it is clear that these studies neither effectively verified cardiovascular outcome indicators nor clarified the essential differences between accumulated exercise and continuous exercise on the premise that baseline physical activity volume and exercise intensity are kept consistent.

Therefore, this study intends to conduct a targeted meta-analysis with the following procedures: first, excluding studies comparing high-intensity accumulated exercise with continuous exercise to ensure strict comparability of exercise intensity; second, systematically searching global studies on the effects of accumulated exercise and continuous exercise on adult cardiovascular health, comprehensively comparing the acute and chronic effects of these two exercise modalities using the TOST, and clarifying their equivalence or unique advantages in improving core cardiometabolic risk indicators such as body composition, blood pressure, blood lipids, and arterial stiffness-related markers. By strictly controlling key variables including exercise intensity and total exercise volume, this study aims to provide more targeted exercise prescription evidence for populations with fragmented time, while filling the methodological flaws and conclusion gaps in existing comparative studies on these two exercise modalities, and offering high-quality evidence-based support for the optimization of exercise intervention strategies at the public health level.

## 2 Methods

This article was written in strict accordance with the requirements of the 2020 PRISMA statement (Page et al., 2021). Prior to conducting any analyses, the research objectives, search terms, and methodologies of this meta-analysis were registered and managed on the PROSPERO platform (registration number: CRD420261281013).

### 2.1 Information sources and search strategy

Literature searches were performed across the Embase, Cochrane Library, PubMed, and Web of Science databases, with all retrieved records imported into EndNote 20 software. The search timeframe spanned from the inception of each database to January 7, 2026. Based on similar previous reviews (Murphy et al., 2009; Murphy et al., 2019; Zhang et al., 2022), the search strategy was formulated as follows (“Skinfold thickness” OR “Skinfold measurement” OR “Triceps skinfold” OR “Subscapular skinfold” OR “Skinfold caliper” OR “Waist circumference” OR “WC” OR “Abdominal circumference” OR “Waist measurement” OR “Obesity” OR “Arterial stiffness index” OR “Cardio-Ankle Vascular Index” OR “ΔCAVI” OR “Systolic Blood Pressure” OR “SBP” OR “Diastolic Blood Pressure” OR “DBP” OR “Pulse Wave Velocity” OR “PWV” OR “Total Cholesterol” OR “TC” OR “Triglycerides” OR “TG” OR “High-Density Lipoprotein Cholesterol” OR “HDL-C” OR “Low-Density Lipoprotein Cholesterol” OR “LDL-C”) AND (“single bout” OR “multiple bout” OR “short” AND “bout” OR “long bout” OR “one bout” OR “repeated bout” OR “continuous” OR “intermittent” OR “accumulate” OR “break” OR “breaks” OR “breaking” OR “interrupting” OR “physical activity pattern” OR “exercise pattern” OR “intervals” OR “alternating” OR “Exercise snack” OR “periodic exercise”) AND (“exercise” OR “sports” OR “resistance training” OR “physical activity” OR “walk” OR “sport” OR “weight training” OR “weightlifting” OR “fitness” OR “running” OR “cycling” OR “swimming”)AND(“randomized controlled trial” OR “random” OR “placebo” OR “single blind” OR “double blind” OR “triple blind”) NOT(“comment” OR “editorial” OR “meta-analysis” OR “practice-guideline” OR “review” OR “letter” OR “journal correspondence” OR “random sample” OR “random digit” OR “random effect” OR “random survey” OR “random regression”). Meanwhile, two researchers (WY and HP) independently conducted full-text screening and cross-verification. Any discrepancies were resolved through discussion with a third researcher to determine the eligibility of the studies. The database search strategies and retrieval results are provided in **Supplementary Table 1**.

### 2.2 Inclusion and exclusion criteria

In this study, literature screening criteria were established following the PICOS framework. Only human trials were included without restrictions on participants’ gender, age or physical health status. Referring to the studies by Murphy et al. (2019) and Yin et al. (2025), the intervention was defined as accumulated exercise consisting of two or more short exercise bouts per day. Strict matching was required between the accumulated exercise group and the control continuous exercise group in terms of total exercise load, duration or energy expenditure, with matching verified based on exercise duration, intensity, MET values or calorie consumption data reported in original articles. Additionally, eligible studies needed to report at least one cardiometabolic surrogate marker related to body composition, blood pressure, blood lipids or arterial stiffness; studies reporting hard clinical cardiovascular endpoints such as myocardial infarction and stroke were excluded. Eligible study designs were limited to parallel randomized controlled trials (RCTs) or crossover RCTs, and all crossover trials must incorporate a washout period to eliminate residual effects from prior interventions. Exclusion criteria were as follows: publications without full-text access, preprints, non-peer-reviewed papers, non-original studies including reviews, grey literature such as conference abstracts, dissertations and unpublished reports, controlled trials with unmatched exercise loads between two groups, non-human basic experiments (animal or cell studies), and articles providing only qualitative descriptions without extractable quantitative effect data.

### 2.3 Data screening and extraction

Upon completion of the literature search, two researchers (WY and HP) extracted the basic bibliographic information and screened the articles based on predefined inclusion and exclusion criteria. The selected literature was imported into EndNote 20 software, where initial duplicate removal was performed, followed by preliminary screening based on title and abstract review. Finally, the full-text articles of the preliminary screened studies were downloaded for eligibility assessment. Data extraction and calculation/transformation were conducted using a customized Microsoft Excel template. The two researchers independently cross-checked the full-text screening results. Any discrepancies were resolved through discussion between the two reviewers.

### 2.4 Risk of bias and quality of methods assessment

For randomized controlled trials (RCTs), the PEDro scale (Maher et al., 2003) was used for quality assessment. The scale comprises 11 items, scored on a 0–10 point basis (Item 1 is not scored, while each of the remaining 10 items is assigned 1 point). Studies with a score of ≥6 were classified as high quality, 4–5 as moderate quality, and ≤3 as low quality. The Cochrane Risk of Bias Tool 2.0 (RoB2) was employed to assess the risk of bias, which evaluates five domains: random sequence generation, allocation concealment, blinding of outcome assessment, incomplete outcome data, and selective outcome reporting.

For non-randomized controlled trials, the Methodological Index for Non-Randomized Studies (MINORS) scale (Slim et al., 2003) was used for quality assessment. The MINORS scale includes 8 items, scored on a 0–2 point scale (0 = not reported, 1 = partially reported, 2 = fully reported), with a total score ranging from 0 to 16. Studies with a total score of ≥12 were considered high quality, 8–11 as moderate quality, and ≤7 as low quality. The Risk Of Bias In Non-randomized Studies - of Interventions (ROBINS-I) tool was used to assess the risk of bias, covering seven domains: confounding, selection of participants, classification of interventions, adherence to intervention protocols, handling of missing data, measurement of outcome variables, and selective reporting of results.

The above assessments were independently performed by two researchers (WY and HP). Discrepancies between researchers were resolved through discussion whenever possible; if no consensus could be reached, a third reviewer (LJ) acted as the arbiter.

### 2.5 Statistical analysis

All meta-analytical statistical calculations were performed using Stata 16.0. Sensitivity analyses were conducted to assess the influence of each individual included study on the pooled overall effect size. Quantitative publication bias detection was implemented via Egger’s linear regression test and Begg’s rank correlation test to identify potential publication bias across studies.

#### 2.5.1 Data preprocessing and correction for repeated measures

Prior to all statistical calculations, standard deviations were derived from standard errors via (Equation 1) if original studies only reported standard errors without standard deviations. In accordance with the guidance of the Cochrane Handbook (Cumpston et al., 2019) (Equation 2) was adopted to calculate the mean within-participant change (MΔ) from pre- to post-intervention for paired data from crossover trials, single-group pre-post controlled trials, and paired RCTs. (Equation 3) was further used to adjust the standard deviation of change (SDΔ). None of the included studies reported the within-participant correlation coefficient r between baseline and follow-up outcomes. Previous meta-analyses focusing on exercise interventions routinely employ r=0.5 as a moderate correlation reference for primary analyses. Therefore, we uniformly adopted r=0.5 to calculate adjusted SDΔ for the primary analysis, and performed sensitivity analyses using r=0.2 and r=0.8 to test how the choice of r affects pooled effect sizes (**Supplementary Table 6**). These correction formulas remove correlated inter-individual variation between baseline and follow-up measurements, address the overestimation of change dispersion produced by conventional independent-sample formulas, and eliminate statistical bias originating from repeated observations of the same participant.

Furthermore, Hedges’ g was chosen as the standardized point estimate of effect size given the generally small sample sizes across included studies, with its calculation outlined in (Equations 4, 5) (McKenzie et al., 2013). Conventional magnitude thresholds are defined as follows: g< 0.2 indicates negligible between-group differences with minimal practical relevance; 0.2 ≤ g< 0.5 represents small differences with limited clinical magnitude; 0.5 ≤ g< 0.8 corresponds to moderate differences with clear practical implications; and g ≥ 0.8 denotes large differences with substantial practical significance. All Hedges’ g values in this meta-analysis were calculated as the mean value of the accumulated exercise group minus the mean value of the continuous exercise group. A positive g indicates higher levels of the corresponding cardiometabolic marker following accumulated exercise, while a negative g denotes lower levels after accumulated exercise. To avoid confusion when interpreting the sign of effect sizes, the clinical meaning of each marker is explicitly stated throughout the results section.

(1) $$SE=SD/\sqrt{\text{n}}$$

(2) $${\text{M}}_{\Delta }={\text{M}}_{post}-{\text{M}}_{pre}$$

(3) $${\text{SD}}_{\Delta }=\sqrt{{\text{SD}}_{\text{pre}}^{2}+{\text{SD}}_{\text{post}}^{2}-\left(2\times \text{r}\times {\text{SD}}_{pre}\times {\text{SD}}_{post}\right)}$$

(4) $$g=\frac{{\text{M}}_{\Delta 1}-{\text{M}}_{\Delta 2}}{{\text{S}}_{\text{p}}}$$

(5) $${\text{S}}_{\text{p}}=\sqrt{\frac{\left({\text{n}}_{1}-1\right){\text{SD}}_{\Delta 1}^{2}+\left({\text{n}}_{2}-1\right){\text{SD}}_{\Delta 2}^{2}}{{\text{n}}_{1}+{\text{n}}_{2}-2}}$$

#### 2.5.2 Selection of pooled effect models

Fixed-effect and random-effects models each possess distinct applicable scenarios and merits for meta-analytic synthesis. However, the included trials recruited highly heterogeneous populations comprising healthy individuals, patients with hypertension, obese adults and participants with diabetes. Additionally, inherent methodological heterogeneity exists between parallel-group trials and crossover repeated-measure trials. To separate statistical characteristics across the two trial designs, subgroup analyses stratified by study design were performed to pool effect sizes separately. Accordingly, the random-effects model was adopted uniformly for all analyses in the present meta-analysis.

#### 2.5.3 Equivalence testing via two one-sided tests

When the pooled meta-analytic effect failed to reach statistical significance, the TOST procedure was applied to judge whether the intergroup difference between accumulated and continuous exercise fell within a trivial range lacking meaningful clinical relevance (Lakens, 2017).

Following the standardized analytical framework proposed by Lakens et al (Lakens, 2017; Lakens et al., 2018), three symmetric equivalence bounds of Hedges’ g (± 0.1, ± 0.2, ± 0.3) were predefined. The ±0.3 threshold served as the core smallest effect size of interest (SESOI), corresponding to the upper limit of small effects under Cohen’s effect size taxonomy. The ±0.1 and ±0.2 bounds were designated as narrow and mild equivalence thresholds respectively for hierarchical robustness verification. Given the absence of field-specific minimal clinically important difference criteria for cardiovascular exercise research, this multi-tiered boundary setting represents a widely accepted compromise within the academic community.

This study clearly distinguishes two easily confused statistical concepts: a P>0.05 from conventional two-tailed tests merely indicates that the available sample lacks sufficient power to reject the null hypothesis of no intergroup difference, which cannot be directly interpreted as equivalent intervention effects. Narrow, mild or medium equivalence can only be claimed if the 90% confidence interval (CI) of the pooled effect size lies entirely within the corresponding predefined bound. If the 90% CI extends beyond the ±0.3 threshold, the TOST result is classified as inconclusive. The full judgment framework and adoption of 90% CIs were grounded in foundational TOST theories (Lakens, 2017; Lakens et al., 2018), which avoids the common statistical fallacy of misclassifying non-significant findings as evidence of equivalence.

## 3 Results

### 3.1 Literature search results

**Figure 1:**
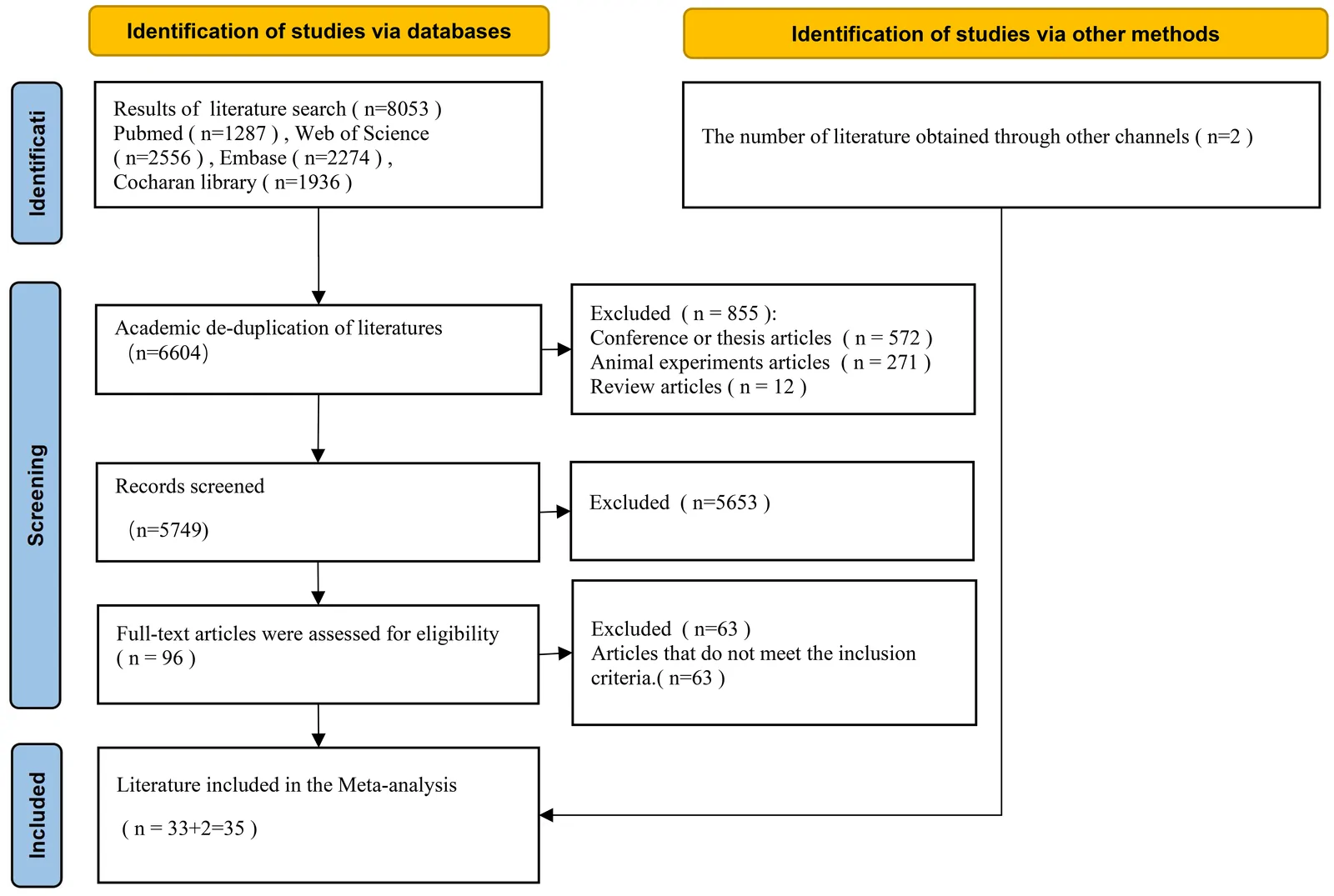
Flow chart of literature screening.

A total of 8,053 studies were retrieved in this research, including 1,287 from the PubMed database, 2,556 from Web of Science, 2,274 from Embase, and 1,936 from the Cochrane Library. Prior to formal screening, 1,449 duplicate studies, 572 conference papers and dissertations, 271 animal experiment articles, and 12 review articles were excluded. Subsequently, 5,653 irrelevant studies were eliminated through title and abstract review. After that, full-text intensive reading and re-evaluation were conducted on the 96 remaining studies; among them, 63 were excluded for failing to meet the predefined inclusion criteria, resulting in the preliminary inclusion of 33 studies (Murphy and Hardman, 1998; Coleman et al., 1999; Jakicic et al., 1999; Murphy et al., 2000; Schmidt et al., 2001; Murphy et al., 2002; Asikainen et al., 2003; Altena et al., 2004; Murtagh et al., 2005; Altena et al., 2006; Park et al., 2006; Quinn et al., 2006; Miyashita, 2008; Miyashita et al., 2008; Miyashita et al., 2011; Serwe et al., 2011; Alizadeh et al., 2013; Peddie et al., 2013; Kim et al., 2014; Manthou et al., 2015; Zheng et al., 2015; Zhou et al., 2015; Miyashita et al., 2016; Reynolds et al., 2016; Bhammar et al., 2017; Chung et al., 2017; Engeroff et al., 2017; Fonseca et al., 2018; Kashiwabara et al., 2018; Madjd et al., 2019; Shambrook et al., 2020; Zhou et al., 2022; Liu et al., 2024). In addition, 2 additional eligible studies (Jakicic et al., 1995; Eguchi et al., 2013) were incorporated via retrospective retrieval and review of relevant research. Thus, a total of 35 studies were finally included in this meta-analysis. Based on differences in intervention duration and effect types, the included studies were categorized into two groups: first, 17 studies (Jakicic et al., 1995; Murphy and Hardman, 1998; Coleman et al., 1999; Jakicic et al., 1999; Schmidt et al., 2001; Murphy et al., 2002; Asikainen et al., 2003; Murtagh et al., 2005; Altena et al., 2006; Quinn et al., 2006; Serwe et al., 2011; Alizadeh et al., 2013; Eguchi et al., 2013; Manthou et al., 2015; Reynolds et al., 2016; Chung et al., 2017; Madjd et al., 2019) investigating long-term intervention effects; second, 18 studies (Murphy et al., 2000; Altena et al., 2004; Park et al., 2006; Miyashita, 2008; Miyashita et al., 2008; Miyashita et al., 2011; Peddie et al., 2013; Kim et al., 2014; Zheng et al., 2015; Zhou et al., 2015; Miyashita et al., 2016; Bhammar et al., 2017; Engeroff et al., 2017; Fonseca et al., 2018; Kashiwabara et al., 2018; Shambrook et al., 2020; Zhou et al., 2022; Liu et al., 2024)analyzing acute effects of single-bout interventions. The detailed literature screening process is illustrated in **Figure 1**.

### 3.2 Basic Characteristics of included studies

A total of 35 studies published between 1995 and 2024 were included in this research, all focusing on the comparison of intervention effects between accumulated exercise and long-duration continuous exercise. All studies ensured that the total exercise duration or total energy expenditure was basically consistent between the two groups to guarantee the comparability of interventions. Except for the study by Peddie et al. (2013) (6 days), all other included randomized crossover controlled trials explicitly reported a washout period of at least 1 week, which effectively avoided the residual effects of prior interventions and ensured the fairness and data reliability of the trials. The population characteristics of the included studies were widely representative, covering different genders and an age range of 18.76–70.5 years, and including healthy individuals as well as patient subgroups such as those with overweight and hypertension, with sample sizes ranging from 8 to 148 participants. Interventions were mainly composed of easy-to-perform and high-safety types such as walking; exercise intensity was standardized in all studies, and the balance of total exercise volume between the two groups was presented in detail in **Supplementary Table 3**.

### 3.3 Risk of bias and quality of methods assessment

A total of 32 randomized controlled trials (RCTs) (Jakicic et al., 1995; Murphy and Hardman, 1998; Coleman et al., 1999; Jakicic et al., 1999; Murphy et al., 2000; Murphy et al., 2002; Asikainen et al., 2003; Murtagh et al., 2005; Park et al., 2006; Quinn et al., 2006; Miyashita, 2008; Miyashita et al., 2008; Miyashita et al., 2011; Serwe et al., 2011; Alizadeh et al., 2013; Eguchi et al., 2013; Peddie et al., 2013; Kim et al., 2014; Manthou et al., 2015; Zheng et al., 2015; Zhou et al., 2015; Miyashita et al., 2016; Reynolds et al., 2016; Bhammar et al., 2017; Chung et al., 2017; Engeroff et al., 2017; Fonseca et al., 2018; Kashiwabara et al., 2018; Madjd et al., 2019; Shambrook et al., 2020; Zhou et al., 2022; Liu et al., 2024) were included in this study. Methodological quality assessment of the included studies using the PEDro scale showed that all studies completed all key outcome assessments, ensured measurement reliability, and reported the statistical significance of between-group differences (**Supplementary Table 4**). The total scores of the 32 RCTs ranged from 4 to 7, indicating an acceptable overall quality: among them, 7 studies (21.88%) were rated as high quality (7 points), 25 studies (78.12%) were rated as moderate quality (4–6 points), and no studies were rated as low quality (≤ 3 points). The Cochrane RoB2 tool was used to assess the risk of bias, with coverage of core domains, clear judgment criteria, and standardized documentation (**Supplementary Figure 1A**). None of the 32 RCTs were assigned an “unclear” judgment, indicating an overall controllable risk of bias: among them, 3 studies (9.4%) were at low risk, 23 studies (71.9%) were at some risk, and 6 studies (18.8%) were at high risk.

A total of 3 non-randomized controlled trials (NRCTs (Schmidt et al., 2001; Altena et al., 2004; Altena et al., 2006) were included in this study. Methodological quality assessment using the MINORS scale showed that 2 studies scored 16 points and 1 study scored 15 points, all of which were rated as high quality (**Supplementary Table**). The Cochrane ROBINS-I tool was used to assess the risk of bias, with complete assessment records and clear reporting (**Supplementary Figure 1B**). None of the 3 NRCTs were identified as having “critical risk”, indicating an overall acceptable risk of bias: among them, 2 studies (66.7%) were at moderate risk, and 1 study (33.3%) was at serious risk.

### 3.4 Sensitivity analysis

Sensitivity analysis was performed using Stata software (**Supplementary Figure 1**), with the metaninf command used to implement the leave-one-out analysis. The pooled effect size was recalculated after excluding each individual study to evaluate the stability of the results. The results showed that after excluding any single study, the pooled effect size and its 95% CI did not change significantly and were highly consistent with the results of the main analysis, indicating that the study results had good reliability and robustness.

### 3.5 Publication bias test

Egger’s Test and Begg’s Test were conducted to assess publication bias, with the results presented in the table. The findings demonstrated that all study results were stable and reliable (**Table 1**).

**Table 1:** Summary of bias test.

Outcome	Egger’s Test	Begg’s Test
	P >| t |(bias)	Pr > |z|
BF	0.665	0.902
LBM	0.448	0.734
BW	0.613	0.822
WC	0.381	0.621
SBP	0.144	0.246
DBP	0.21	0.235
TC	0.638	0.707
TG	0.21	0.548
HDL - C	0.634	1
LDL - C	0.336	0.308
S-FTG	0.13	0.452
S-TG-iAUC	0.982	1
S-SBP	0.762	0.381
S-DBP	0.087	0.827
S-ASI	0.057	0.107

BF, Body Fat; LBM, Lean Body Mass; BW, Body Weight; WC, Waist Circumference; SBP, Systolic Blood Pressure; DBP, Diastolic Blood Pressure; TC, Total Cholesterol; TG, Triglyceride; HDL-C, High-Density Lipoprotein Cholesterol; LDL-C, Low-Density Lipoprotein Cholesterol; S-FTG, Single-exercise Fasting Triglyceride; S-TG-iAUC, Single-exercise Incremental Area Under the Curve of Triglyceride; S-SBP, Single-exercise Systolic Blood Pressure; S-DBP, Single-exercise Diastolic Blood Pressure; S-ASI, Single-exercise Arterial Stiffness-Associated Markers.

### 3.6 Primary analysis results

#### 3.6.1 Comparison of long-term effects between accumulated exercise and continuous exercise

BF yielded a pooled Hedges’ g = −0.02. A negative g value indicated lower BF in the AE group relative to CE, with lower BF corresponding to superior intervention benefits. The 95% CI crossed zero (95% CI: −0.26, 0.22; 90% CI: −0.22, 0.19; P = 0.891,I²= 15.9%), revealing no statistically significant between-group difference. The entire 90% CI fell within the ±0.3 medium equivalence bound, and TOST confirmed medium equivalence, indicating no clinically meaningful distinction between AE and CE for BF (**Table 2**).

**Table 2:** Summary of the comparison results of long-term effects between accumulated exercise and continuous exercise.

Outcome	K	n	Hedges’ g	(95% CI)	I^2^	P-value	(90% CI)	Equivalence tests
Body composition indicators								
BF	8	352	-0.02	(-0.26, 0.22)	15.9%	0.891	(-0.22, 0.19)	medium equivalence
LBM	4	257	0.03	(-0.22, 0.27)	0%	0.834	(-0.18, 0.23)	medium equivalence
BW	16	460	-0.2	(-0.39,-0.01)	0%	0.035	-	-
WC	15	589	0.01	(-0.16, 0.17)	0%	0.943	(-0.13, 0.14)	narrow equivalence
Cardiometabolic risk markers								
SBP	13	443	-0.18	(-0.17, 0.52)	66.7%	0.315	(-0.11, 0.46)	NA
DBP	15	508	0.16	(-0.10, 0.42)	50.7%	0.232	(-0.06, 0.38)	NA
TC	6	219	-0.03	(-0.30, 0.23)	0%	0.801	(-0.26, 0.19)	medium equivalence
TG	7	242	-0.03	(-0.28, 0.22)	0%	0.801	(-0.25, 0.18)	medium equivalence
HDL - C	7	243	0.09	(-0.16, 0.35)	0%	0.475	(-0.12, 0.31)	NA
LDL - C	4	156	-0.10	(-0.41, 0.22)	0%	0.548	(-0.36, 0.17)	NA

BF, Body Fat; LBM, Lean Body Mass; BW, Body Weight; WC, Waist Circumference; SBP, Systolic Blood Pressure; DBP, Diastolic Blood Pressure; TC, Total Cholesterol; TG, Triglyceride; HDL-C, High-Density Lipoprotein Cholesterol; LDL-C, Low-Density Lipoprotein Cholesterol.

LBM yielded a pooled Hedges’ g = 0.03. A positive g value indicated higher LBM in the AE group relative to CE, with higher LBM corresponding to superior intervention benefits. The 95% CI crossed zero (95% CI: −0.22, 0.27; 90% CI: −0.18, 0.23; P = 0.834, I² = 0%), revealing no statistically significant between-group difference. The entire 90% CI fell within the ±0.3 medium equivalence bound, and TOST confirmed medium equivalence, demonstrating no material difference in LBM improvement between AE and CE (**Table 2**).

WC yielded a pooled Hedges’ g = 0.01. A positive g value indicated slightly higher WC in the AE group relative to CE, with lower WC corresponding to superior intervention benefits. The 95% CI crossed zero (95% CI: −0.16, 0.17; 90% CI: −0.13, 0.14; P = 0.943, I² = 0%), revealing no statistically significant between-group difference. The entire 90% CI fell within the ±0.1 narrow equivalence bound, and TOST confirmed narrow equivalence, showing highly comparable WC regulation effects of AE and CE (**Table 2**).

BW yielded a pooled Hedges’ g = −0.20. A negative g value indicated lower BW in the AE group relative to CE, with lower BW corresponding to superior intervention benefits. The 95% CI did not cross zero (95% CI: −0.39, −0.01; P = 0.035, I²= 0%), presenting a statistically significant between-group difference and suggesting a minor statistical advantage of AE for BW management (**Table 2**).

TC yielded a pooled Hedges’ g = −0.03. A negative g value indicated lower TC in the AE group relative to CE, with lower TC associated with reduced cardiovascular risk. The 95% CI crossed zero (95% CI: −0.30, 0.23; 90% CI: −0.26, 0.19; P = 0.801, I² = 0%), revealing no statistically significant between-group difference. The entire 90% CI fell within the ±0.3 medium equivalence bound, and TOST confirmed medium equivalence, meaning AE and CE exerted no clinically distinct regulatory effects on TC (**Table 2**).

TG yielded a pooled Hedges’ g = −0.03. A negative g value indicated lower TG in the AE group relative to CE, with lower TG representing better lipid metabolic status. The 95% CI crossed zero (95% CI: −0.28, 0.22; 90% CI: −0.25, 0.18; P = 0.801, I² = 0%), revealing no statistically significant between-group difference. The entire 90% CI fell within the ±0.3 medium equivalence bound, and TOST confirmed medium equivalence, showing no substantive distinction in long-term lipid-lowering effects between AE and CE (**Table 2**).

SBP yielded a pooled Hedges’ g = −0.18. A negative g value indicated lower SBP in the AE group relative to CE, with lower BP associated with greater cardiovascular benefits. The 95% CI crossed zero (95% CI: −0.17, 0.52; 90% CI: −0.11, 0.46; P = 0.315, I² = 66.7%), revealing no statistically significant between-group difference. The 90% CI extended beyond the ±0.3 equivalence range, and TOST returned inconclusive results; current evidence cannot verify whether AE and CE produce equivalent SBP regulation (**Table 2**).

DBP yielded a pooled Hedges’ g = 0.16. A positive g value indicated higher DBP in the AE group relative to CE, with lower DBP corresponding to superior intervention benefits. The 95% CI crossed zero (95% CI: −0.10, 0.42; 90% CI: −0.06, 0.38; P = 0.232, I²= 50.7%), revealing no statistically significant between-group difference. The 90% CI spanned the upper and lower limits of the ±0.3 equivalence bound, and TOST returned inconclusive results; equivalence between AE and CE for DBP could not be confirmed (**Table 2**).

HDL-C yielded a pooled Hedges’ g = 0.09. A positive g value indicated higher HDL-C in the AE group relative to CE, with higher HDL-C conferring stronger cardiovascular protection. The 95% CI crossed zero (95% CI: −0.16, 0.35; 90% CI: −0.12, 0.31; P = 0.475, I^2^ = 0%), revealing no statistically significant between-group difference. The 90% CI extended beyond the ±0.3 equivalence range, and TOST returned inconclusive results (**Table 2**).

LDL-C yielded a pooled Hedges’ g = −0.10. A negative g value indicated lower LDL-C in the AE group relative to CE, with lower LDL-C linked to reduced cardiovascular risk. The 95% CI crossed zero (95% CI: −0.41, 0.22; 90% CI: −0.36, 0.17; P = 0.548, I² = 0%), revealing no statistically significant between-group difference. The 90% CI spanned the upper and lower limits of the ±0.3 equivalence bound, and TOST also returned inconclusive results (**Table 2**).

#### 3.6.2 Comparison of acute effects between accumulated exercise and continuous exercise

The pooled Hedges’ g = S-FTG was −0.08. A negative value indicated lower S-FTG in the AE group, and lower S-FTG reflected superior lipid metabolism. The 95% CI (−0.44, 0.28) crossed zero, showing no statistically significant intergroup difference (P = 0.648, I² = 0%). Its 90% CI (−0.39, 0.22) exceeded both upper and lower ±0.3 equivalence bounds, with TOST yielding inconclusive results. Current evidence neither confirmed equivalent regulatory effects of AE and CE on S-FTG nor identified clinically meaningful intergroup discrepancies (**Table 3**).

**Table 3:** Summary of the comparison results of acute effects between accumulated exercise and continuous exercise.

Outcome	Group	K	n	Hedges’ g	(95% CI)	I^2^	P-value	(90% CI)	Equivalence tests
S-FTG	-	6	120	-0.08	(-0.44, 0.28)	0%	0.648	(-0.39, 0.22)	NA
S-TG-iAUC	-	8	324	0.24	(0.02,0.46)	0%	0.03	-	-
S-SBP	10-20min	5	230	-0.61	(-1.03, -0.18)	47.1%	0.005	-	-
	20-40min	3	140	-0.47	(-0.97, 0.03)	49.2%	0.065	(-0.89, -0.05)	NA
	40-60min	3	98	-0.10	(-0.50, 0.30)	0%	0.639	(-0.43, 0.24)	NA
	24h	3	70	-0.16	(-0.84, 0.52)	49.9%	0.637	(-0.73, 0.41)	NA
	Overall	14	538	-0.38	(-0.61, -0.15)	33%	0.001	-	-
S-DBP	10-20min	5	230	-0.14	(-0.43, 0.15)	0%	0.335	(-0.38, 0.10)	NA
	20-40min	3	140	-0.61	(-1.11, -0.10)	49.8%	0.018	-	-
	40-60min	3	98	0.02	(-0.38, 0.42)	0%	0.915	(-0.31, 0.36)	NA
	24h	3	70	0.10	(-0.37, 0.57)	0%	0.676	(-0.28, 0.48)	NA
	Overall	14	538	-0.19	(-0.39, 0.02)	23.4%	0.077	(-0.38, 0.01)	NA
S-ASI	PWV	1	20	-0.76	(-1.67, 0.16)	-	0.105	(-1.49, -0.02)	NA
	ASIβ	2	120	0.19	(-0.26, 0.63)	34.5%	0.410	(-0.20, 0.57)	NA
	⊿CAVI	8	284	-0.56	(-0.96, -0.16)	60.1%	0.006	-	-
	Overall	11	424	-0.41	(-0.77, -0.04)	68.2%	0.028	(-0.71, -0.10)	NA

S-FTG, Single-exercise Fasting Triglyceride; S-TG-iAUC, Single-exercise Incremental Area Under the Curve of Triglyceride; S-SBP, Single-exercise Systolic Blood Pressure; S-DBP, Single-exercise Diastolic Blood Pressure,S-ASI, Single-exercise Arterial Stiffness-Associated Markers.

The pooled Hedges’ g = S-TG-iAUC was 0.24. A positive value denoted higher postprandial triglyceride exposure in the AE group, and elevated postprandial lipid load impaired lipid metabolism. The 95% CI (0.02, 0.46) did not cross zero, indicating a statistically significant intergroup difference (P = 0.03, I² = 0%). Nevertheless, this effect fell within the small effect range with trivial numerical disparity, which only represented minor statistical distinction and possessed limited clinical discriminatory value. Conclusions should be interpreted cautiously with consideration of the sample size of included studies (**Table 3**).

S-SBP exhibited time-stratified characteristics: the 10–20 min subgroup had a Hedges’ g = −0.61. A negative value represented lower S-SBP in the AE group, and reduced Blood Pressure benefited cardiovascular health. Its 95% CI (−1.03, −0.18) excluded zero, revealing a statistically significant difference (P = 0.005) with moderate heterogeneity (I² = 47.1%). The 20–40 min subgroup had a 90% CI (−0.89, −0.05) with only a marginal statistical trend (P = 0.065). The 95% CIs of the 40–60 min and 24 h subgroups all crossed zero without statistical significance, and corresponding TOST results were inconclusive. The overall pooled Hedges’ g for S-SBP was −0.38; the negative value meant lower S-SBP in the AE group. Its 95% CI (−0.61, −0.15) did not cross zero, demonstrating a statistically significant overall difference (P = 0.001) with low heterogeneity (I²= 33%). However, such disparity only existed within a short post-exercise window as a transient response, which cannot be regarded as evidence of sustained long-term antihypertensive superiority of AE (**Table 3**).

Statistical significance in S-DBP was only observed in the 20–40 min subgroup, with a Hedges’ g = −0.61. The negative value indicated lower S-DBP in the AE group, and its 95% CI (−1.11, −0.10) excluded zero (P = 0.018). The 95% CIs of all other time subgroups and the overall pooled estimate crossed zero without statistical significance (overall P = 0.077). The 90% CIs of all time points extended beyond the ±0.3 equivalence interval, and all TOST outcomes were inconclusive. Minor statistical gaps merely emerged within an extremely short time window, failing to distinguish clinical superiority between AE and CE for DBP regulation (**Table 3**).

The overall pooled Hedges’ g = S-ASI was −0.41. A negative value represented lower arterial stiffness markers in the AE group, corresponding to better vascular elasticity. Its 95% CI (−0.77, −0.04) excluded zero with a statistically significant difference (P = 0.028), and the 90% CI was (−0.71, −0.10). The ΔCAVI subgroup had a Hedges’ g = −0.56 with a 95% CI (−0.96, −0.16) and significant statistical difference (P = 0.006), yet this subgroup presented high heterogeneity (I² = 60.1%). The 95% CIs of PWV and ASIβ subgroups both crossed zero without statistical significance, and TOST tests returned inconclusive results. Although minor overall statistical disparities were detected, high between-study heterogeneity undermined the generalizability of these findings. Such results should not be overinterpreted as stable superior vascular improvements induced by AE (**Table 3**).

## 4 Discussion

This meta-analysis strictly matched exercise intensity and total volume between AE and CE, and adopted the TOST procedure to simultaneously distinguish intergroup differences and equivalence status, systematically elucidating acute and chronic cardiometabolic regulatory profiles induced by the two exercise modalities in adults. Compared with prior reviews, the present analysis incorporated outcomes from both single-bout acute sessions and long-term regular training, filling the gap that previous syntheses failed to comprehensively integrate cardiovascular surrogate markers.

### 4.1 Long-term effects of accumulated exercise versus continuous exercise on cardiometabolic indications

Chronic regular exercise serves as a core strategy for cardiovascular disease management, with its benefits mediated by chronic physiological remodeling in response to exercise stimuli (Kim et al., 2025). We stratified analyses across three categories: body composition, blood pressure, and blood lipids. Overall, most endpoints satisfied equivalence criteria, with only small-magnitude statistical differences observed for BW.

#### 4.1.1 Long-term effects of accumulated exercise versus continuous exercise on body composition

The 90% CIs for BF, LBM and WC all fell entirely within the predefined equivalence margins. TOST confirmed equivalent long-term regulatory efficacy of AE and CE for these three markers. Only BW yielded a pooled Hedges’ g of −0.20, reflecting a minor statistical disparity between modalities. By standard effect size thresholds, this value fell within the trivial effect range, indicating minimal discriminative capacity for BW between AE and CE.

In addition, sensitivity tests were conducted by substituting different within-participant correlation coefficients r into (Equation 3) in accordance with the Cochrane Handbook. When r=0.2, Stata output a pooled Hedges’ g of −0.18 for BW, with a corresponding 95% CI of (0.368, 0.000). Given the upper bound approximated an infinitesimal positive value close to zero, the pooled intergroup effect for BW was judged statistically non-significant under the r=0.2 assumption.

These findings align with the results reported by Murphy et al. (2019). A superficial mechanistic interpretation, grounded in standardized intervention conditions across included trials, posits that segmented activity breaks prolonged sedentary time and theoretically elevates daily total energy expenditure to a small degree (Wan et al., 2025; Zhou et al., 2025), conferring a modest BW-lowering advantage to AE. Nevertheless, this deduction remains only a basic physiological inference unsupported by continuous energy-monitoring data from the included studies; therefore, no in-depth mechanistic speculation is advanced herein. In summary, AE and CE produce statistically indistinguishable long-term effects on core obesity markers including BF and WC, with the trivial BW disparity lacking clinical discriminatory value. This indicates the two regimens can act as interchangeable long-term interventions. For populations unable to sustain unbroken prolonged exercise, the subtle BW benefit of AE carries practical value for improving exercise time adherence.

#### 4.1.2 Long-term effects of accumulated exercise versus continuous exercise on blood pressure

Prior investigations have yielded conflicting findings regarding long-term hypotensive effects of the two exercise regimens (Murphy et al., 2009). The overall 95% CIs of SBP and DBP crossed zero, and their 90% CIs exceeded the ±0.3 equivalence bound, yielding inconclusive TOST outcomes. Thus, existing evidence cannot confirm or refute intergroup differences in long-term Blood Pressure reduction. Moderate-to-high heterogeneity was observed in primary pooled analyses. After excluding Manthou et al. (2015) and Jakicic et al. (1995), I^2 for SBP decreased to 0% and met the medium equivalence criterion (90% CI: −0.19, 0.28). Exclusion of Manthou et al. (2015) alone reduced I² for DBP to 0%, satisfying small equivalence bounds (90% CI: −0.12, 0.19), which indicated these two trials were the primary contributors to overall heterogeneity in Blood Pressure outcomes.Such heterogeneity revealed substantial confounding from divergent intervention protocols and varied participant baseline characteristics across included studies.

The pooled evidence lacked sufficient stability to support conclusions of superior long-term hypotensive efficacy for either regimen. Vasodilation and other Blood Pressure-lowering physiological pathways represent universal exercise-related mechanisms; quantitative vascular function data were unavailable in this meta-analysis, so extended mechanistic speculation was avoided. Statistical results alone indicated comparable Blood Pressure regulation between AE and CE under strictly standardized supervision and matched exercise loads, while available sample data failed to generate robust conclusions under real-world unsupervised exercise conditions.

#### 4.1.3 Long-term effects of accumulated exercise versus continuous exercise on blood lipids

All core lipid markers exhibited I^2^ = 0%, indicating zero between-study heterogeneity. TOST confirmed medium equivalence for pooled TC and TG. The 90% CIs of HDL-C and LDL-C extended beyond the ±0.3 equivalence range, resulting in inconclusive equivalence test results.

Collectively, long-term steady-state lipid regulation was primarily determined by total cumulative exercise volume over the training cycle, with weak dependence on single-session time-splitting patterns. Weekly consistent total activity volume and intensity sustained comparable long-term HDL-C synthesis and LDL-C clearance, regardless of whether exercise was split into multiple bouts. All included trials matched total energy expenditure between groups, eliminating confounding from unequal exercise volume and producing equivalent effects for TC and TG. Failure to reach equivalence thresholds for HDL-C and LDL-C was tentatively attributed to limited sample sizes and divergent baseline lipid profiles across participants. Deep mechanistic deductions were not performed due to data constraints, and statistical results only demonstrated that consistent long-term differentiation of these two lipid markers could not be achieved between AE and CE.

### 4.2 Acute effects of accumulated exercise versus continuous exercise on cardiometabolic indicators

All acute endpoints in this analysis only captured instantaneous physiological responses following a single exercise session. Although several indicators yielded statistically significant differences, the corresponding effect sizes were merely small to moderate. Subgroups of SBP and arterial stiffness presented moderate-to-high heterogeneity, and all detected disparities were transient without evidence of persistent long-term benefits. Via time-stratified subgroup analyses and quantitative TOST assessment, this study delineated distinct acute response patterns between AE and CE, providing empirical references for the design of future single-bout exercise trials.

#### 4.2.1 Acute effects of accumulated exercise versus continuous exercise on blood lipids

The pooled 95% CI of S-FTG crossed zero, indicating no statistical intergroup difference. Its 90% CI exceeded both upper and lower ±0.3 equivalence thresholds, leading to inconclusive TOST results. Current datasets cannot identify material distinctions in fasting baseline lipid levels between single-bout AE and CE. The pooled Hedges’ g of S-TG-iAUC reached 0.24 with a non-zero 95% CI, demonstrating statistical significance, yet this magnitude fell within the small effect range.

Objective statistical inference suggests that split equal-volume exercise repeatedly activates lipolysis and transiently elevates postprandial lipid exposure, consistent with prior work by Freese et al. (2014) and Pearson et al. (2022). Nevertheless, such fluctuations are confined exclusively to the postprandial window after one acute session and lack long-term follow-up data confirming sustained metabolic shifts; only minor numerical gaps between groups can be objectively described.

#### 4.2.2 Acute effects of accumulated exercise versus continuous exercise on blood pressure

Statistical separation for SBP was only observed in the 10–20 min post-exercise subgroup, with merely marginal trends at 20–40 min and no intergroup disparities at 40–60 min or 24 h follow-up. While the overall pooled estimate reached statistical significance, this acute vascular reaction was short-lived. The 10–20 min subgroup carried moderate heterogeneity (I^2^ = 47.1%), as baseline Blood Pressure and split session duration modulated the magnitude of transient hypotension. Significant DBP differences were limited to the narrow 20–40 min window; all remaining time-stratified subgroups and the overall pooled analysis returned non-significant 95% CIs. TOST yielded inconclusive outcomes across all time points.

These Blood Pressure-lowering acute responses exhibited obvious time-window dependence, with transient vascular relaxation fading as post-exercise observation extended. All trials matched total single-session exercise volume to eliminate volume confounding. Minor short-term Blood Pressure gaps were hypothesized to arise from cumulative residual vasodilatory effects induced by segmented activity (Fonseca et al., 2018; Tsoi et al., 2024). Such transient reactions were heavily modulated by participants’ baseline Blood Pressure and bout segmentation schemes, which explains the moderate subgroup heterogeneity observed. Based solely on the available statistical outputs, these brief small-magnitude differences cannot support claims of durable antihypertensive superiority, and no in-depth mechanistic speculation regarding vascular regulation is advanced here.

#### 4.2.3 Acute effects of accumulated exercise versus continuous exercise on arterial stiffness

The pooled overall estimate for arterial stiffness yielded a statistically significant difference. The 95% CIs of the PWV and ASIβ subgroups both crossed zero, with all corresponding TOST results inconclusive. The ΔCAVI subgroup returned a Hedges’ g of −0.56 with significant intergroup disparity, accompanied by high heterogeneity (I^2 = 60.1%). After excluding the arms reported by Zheng (AE60) (Zheng et al., 2015) and Zhou (ISS) (Zhou et al., 2015), I^2 for the ΔCAVI subgroup fell to 0%, while the effect size remained moderate (Hedges’ g = −0.54, 95% CI: −0.83, −0.24). This indicated that heterogeneity originated from divergent designs of exercise bouts and rest intervals.

Combining pooled outcomes and trial protocols of Zheng et al. (2015) and Zhou et al. (2015), facilitates interpretation of the high heterogeneity within the ΔCAVI subgroup. Zheng et al. (2015) adopted protocols with 60-minute long rest intervals, where immediate vasodilatory effects triggered by the preceding bout almost fully dissipated, failing to generate cumulative physiological responses. By contrast, Zhou et al. (2015), implemented a segmented regimen of four 5-minute bouts separated by 5-minute short rest periods, which sustained short-term vascular improvements. However, this bout partitioning scheme differed substantially from most included trials, amplifying between-study dispersion. Further subgroup comparisons conducted by Zhou et al. (2015), revealed that three 10-minute accumulated bouts with 60-minute long rest intervals still induced transient vascular benefits, yet outcomes differed from two 15-minute bouts with extended rest. This further demonstrated that combinations of single-bout duration and splitting frequency alter the magnitude of acute vascular responses.

The above comparisons are merely superficial deductions grounded in raw observational data from primary studies, without in-depth exploration of underlying vascular physiological mechanisms. Bout duration, splitting frequency, and inter-bout rest intervals are identified as critical confounding variables driving fluctuations in pooled arterial stiffness results. Future standardized primary trials should adopt unified segmented intervention protocols to define optimal matching regimens, thereby improving the robustness and clinical translational value of evidence in this field.

### 4.3 Limitations

This systematic review and meta-analysis evaluated the acute and chronic effects of accumulated versus continuous exercise on cardiometabolic risk biomarkers in adults, yet several methodological and design limitations remain. First, the sample sizes of included studies were unbalanced, with a small number of trials available for some lipid and arterial stiffness subgroups, resulting in low statistical power. Minor statistical differences were vulnerable to random error and could not support robust inferences. Second, moderate to high between-study heterogeneity was detected for blood pressure and arterial stiffness outcomes, driven by confounding variables including intervention duration, inter-bout rest intervals, participants’ baseline health status, and exercise supervision strategies, which introduced high uncertainty into pooled effect estimates. Third, only English-language databases were searched, with no inclusion of Chinese grey literature, leading to potential language-related publication bias; small studies reporting non-significant findings may have been omitted, further weakening the reliability of the overall evidence. Fourth, only surrogate metabolic markers were analyzed without hard clinical endpoints of cardiovascular disease, restricting the generalizability of the present results.

## 5 Conclusions

When matched for total exercise load, accumulated exercise achieves statistical equivalence with continuous exercise for long-term regulation of multiple cardiometabolic risk biomarkers including body fat, lean body mass, waist circumference, total cholesterol and triglycerides, with only a minor statistically significant advantage observed in body weight control that carries limited clinical value. For single acute exercise sessions, accumulated exercise only generates transient beneficial changes in systolic blood pressure, arterial stiffness and postprandial lipid levels within specific post-exercise time windows. The present findings demonstrate that the two exercise modalities are interchangeable intervention options, and clinicians can formulate individualized exercise prescriptions according to participants’ time adherence profiles. Future research should further investigate the optimal intervention protocols for accumulated exercise and the efficacy of multi-factor combined intervention strategies.
