# Supplementary material for: Acute and chronic effects of cumulative vs. continuous physical activity on cardiovascular metabolic risk biomarkers in adults: a systematic review and meta-analysis
Source: Front Physiol. 2026 Aug 28;17:1804202. doi: 10.3389/fphys.2026.1804202 (PMC13563650; doi:10.3389/fphys.2026.1804202)
Supplement: Supplementary file 1 [file Supplementaryfile1.docx]

**Supplementary Table 1( PRISMA 2020 checklist )**

| **Section and Topic** | | **Item #** | | **Checklist item** | **Location where item is reported** |
| --- | --- | --- | --- | --- | --- |
| **TITLE** | | | | |  |
| Title | 1 | | Identify the report as a systematic review. | | Title page |
| **ABSTRACT** | | | | |  |
| Abstract | 2 | | See the PRISMA 2020 for Abstracts checklist. | | Abstract |
| **INTRODUCTION** | | | | |  |
| Rationale | 3 | | Describe the rationale for the review in the context of existing knowledge. | | Introduction |
| Objectives | 4 | | Provide an explicit statement of the objective(s) or question(s) the review addresses. | | Introduction |
| **METHODS** | | | | |  |
| Eligibility criteria | 5 | | Specify the inclusion and exclusion criteria for the review and how studies were grouped for the syntheses. | | Methods 1.2 Inclusion and Exclusion Criteria |
| Information sources | 6 | | Specify all databases, registers, websites, organisations, reference lists and other sources searched or consulted to identify studies. Specify the date when each source was last searched or consulted. | | Methods 1.1 Information Sources and Search Strategy; Supplementary Table 2 |
| Search strategy | 7 | | Present the full search strategies for all databases, registers and websites, including any filters and limits used. | | Methods 1.1 Information Sources and Search Strategy; Supplementary Table 2 |
| Selection process | 8 | | Specify the methods used to decide whether a study met the inclusion criteria of the review, including how many reviewers screened each record and each report retrieved, whether they worked independently, and if applicable, details of automation tools used in the process. | | Methods 1.3 Data Screening and Extraction |
| Data collection process | 9 | | Specify the methods used to collect data from reports, including how many reviewers collected data from each report, whether they worked independently, any processes for obtaining or confirming data from study investigators, and if applicable, details of automation tools used in the process. | | Methods 1.3 Data Screening and Extraction; Supplementary Tables 3–6 |
| Data items | 10a | | List and define all outcomes for which data were sought. Specify whether all results that were compatible with each outcome domain in each study were sought (e.g. for all measures, time points, analyses), and if not, the methods used to decide which results to collect. | | Methods 1.5 Statistical Analysis; Tables 3–4 |
|  | 10b | | List and define all other variables for which data were sought (e.g. participant and intervention characteristics, funding sources). Describe any assumptions made about any missing or unclear information. | | Methods 1.2 Inclusion and Exclusion Criteria; Supplementary Table 3 |
| Study risk of bias assessment | 11 | | Specify the methods used to assess risk of bias in the included studies, including details of the tool(s) used, how many reviewers assessed each study and whether they worked independently, and if applicable, details of automation tools used in the process. | | Methods 1.4 Risk of Bias and Quality of Methods Assessment; Supplementary Tables 4–5; Supplementary Figure 1A–1B |
| Effect measures | 12 | | Specify for each outcome the effect measure(s) (e.g. risk ratio, mean difference) used in the synthesis or presentation of results. | | Methods 1.5.1 Data Preprocessing and Correction for Repeated Measures |
| Synthesis methods | 13a | | Describe the processes used to decide which studies were eligible for each synthesis (e.g. tabulating the study intervention characteristics and comparing against the planned groups for each synthesis (item #5)). | | Methods 1.2 Inclusion and Exclusion Criteria |
|  | 13b | | Describe any methods required to prepare the data for presentation or synthesis, such as handling of missing summary statistics, or data conversions. | | Methods 1.5.1 Data Preprocessing and Correction for Repeated Measures |
|  | 13c | | Describe any methods used to tabulate or visually display results of individual studies and syntheses. | | Results; Supplementary Figures 3A–3O |
|  | 13d | | Describe any methods used to synthesize results and provide a rationale for the choice(s). If meta-analysis was performed, describe the model(s), method(s) to identify the presence and extent of statistical heterogeneity, and software package(s) used. | | Methods 1.5.2 Selection of Pooled Effect Models |
|  | 13e | | Describe any methods used to explore possible causes of heterogeneity among study results (e.g. subgroup analysis, meta-regression). | | Results 2.6.1–2.6.2; Discussion 3.1.2, 3.2.3 |
|  | 13f | | Describe any sensitivity analyses conducted to assess robustness of the synthesized results. | | Methods 1.5.1; Results 2.4 Sensitivity Analysis; Supplementary Table 6; Supplementary Figures 2A–2O |
| Reporting bias assessment | 14 | | Describe any methods used to assess risk of bias due to missing results in a synthesis (arising from reporting biases). | | Methods 1.5 Statistical Analysis; Results 2.5 Publication Bias Test; Table 2 |
| Certainty assessment | 15 | | Describe any methods used to assess certainty (or confidence) in the body of evidence for an outcome. | | Methods 1.5.3 Equivalence Testing via Two One-Sided Tests (TOST); Results Tables 3–4 |
| **RESULTS** | | | | |  |
| Study selection | 16a | | Describe the results of the search and selection process, from the number of records identified in the search to the number of studies included in the review, ideally using a flow diagram. | | Results 2.1 Literature Search Results; Figure 1 |
|  | 16b | | Cite studies that might appear to meet the inclusion criteria, but which were excluded, and explain why they were excluded. | | Methods 1.2 Inclusion and Exclusion Criteria; Results 2.1 Literature Search Results |
| Study characteristics | 17 | | Cite each included study and present its characteristics. | | Results 2.2 Basic Characteristics of Included Studies; Supplementary Table 3 |
| Risk of bias in studies | 18 | | Present assessments of risk of bias for each included study. | | Results 2.3 Risk of Bias and Quality of Methods Assessment; Supplementary Tables 4–5; Supplementary Figure 1A–1B |
| Results of individual studies | 19 | | For all outcomes, present, for each study: (a) summary statistics for each group (where appropriate) and (b) an effect estimate and its precision (e.g. confidence/credible interval), ideally using structured tables or plots. | | Results 2.6 Primary Analysis Results; Tables 3–4; Supplementary Figures 3A–3O |
| Results of syntheses | 20a | | For each synthesis, briefly summarise the characteristics and risk of bias among contributing studies. | | Results 2.2–2.3 |
|  | 20b | | Present results of all statistical syntheses conducted. If meta-analysis was done, present for each the summary estimate and its precision (e.g. confidence/credible interval) and measures of statistical heterogeneity. If comparing groups, describe the direction of the effect. | | Results 2.6 Primary Analysis Results; Tables 3–4 |
|  | 20c | | Present results of all investigations of possible causes of heterogeneity among study results. | | Results 2.6.1–2.6.2; Discussion 3.1.2, 3.2.3 |
|  | 20d | | Present results of all sensitivity analyses conducted to assess the robustness of the synthesized results. | | Results 2.4 Sensitivity Analysis; Supplementary Table 6; Supplementary Figures 2A–2O |
| Reporting biases | 21 | | Present assessments of risk of bias due to missing results (arising from reporting biases) for each synthesis assessed. | | Results 2.5 Publication Bias Test; Table 2 |
| Certainty of evidence | 22 | | Present assessments of certainty (or confidence) in the body of evidence for each outcome assessed. | | Methods 1.5.3 TOST; Results Tables 3–4 |
| **DISCUSSION** | | | | |  |
| Discussion | 23a | | Provide a general interpretation of the results in the context of other evidence. | | Discussion 3.1–3.2 |
|  | 23b | | Discuss any limitations of the evidence included in the review. | | Discussion 3.3 Limitations |
|  | 23c | | Discuss any limitations of the review processes used. | | Discussion 3.3 Limitations |
|  | 23d | | Discuss implications of the results for practice, policy, and future research. | | Discussion 3.4 Conclusions |
| **OTHER INFORMATION** | | | | |  |
| Registration and protocol | 24a | | Provide registration information for the review, including register name and registration number, or state that the review was not registered. | | Methods opening paragraph; PROSPERO registration statement |
|  | 24b | | Indicate where the review protocol can be accessed, or state that a protocol was not prepared. | | Methods opening paragraph |
|  | 24c | | Describe and explain any amendments to information provided at registration or in the protocol. | | Not applicable |
| Support | 25 | | Describe sources of financial or non-financial support for the review, and the role of the funders or sponsors in the review. | | Funding section |
| Competing interests | 26 | | Declare any competing interests of review authors. | | Conflict of Interest section |
| Availability of data, code and other materials | 27 | | Report which of the following are publicly available and where they can be found: template data collection forms; data extracted from included studies; data used for all analyses; analytic code; any other materials used in the review. | | Supplementary Tables and Figures; Manuscript raw analysis statements |

*From:* Page MJ, McKenzie JE, Bossuyt PM, Boutron I, Hoffmann TC, Mulrow CD, et al. The PRISMA 2020 statement: an updated guideline for reporting systematic reviews. BMJ 2021;372:n71. doi: 10.1136/bmj.n71

**Supplementary Table 2 Search strategy	( 1.7.2026 )**

| **Data** | **Query** | **Results** |
| --- | --- | --- |
| **Web of Science** | TS=(Skinfold thickness OR skinfold measurement OR triceps skinfold OR subscapular skinfold OR skinfold caliper OR Waist circumference OR WC OR abdominal circumference OR waist measurement OR obesity or or Arterial stiffness index or Cardio-Ankle Vascular Index or ΔCAVI or Systolic Blood Pressure OR SBP OR Diastolic Blood Pressure OR DBP OR Pulse Wave Velocity OR PWV OR Total Cholesterol OR TC OR Triglycerides OR TG OR High-Density Lipoprotein Cholesterol OR HDL-C OR Low-Density Lipoprotein Cholesterol OR LDL-C) AND TS=(single bout OR multiple bout OR (short AND bout ) OR long bout OR one bout OR repeated bout OR continuous OR intermittent OR accumulate OR break OR breaks OR breaking OR interrupting OR physical activity pattern OR exercise pattern OR intervals OR alternating OR Exercise snack OR periodic exercise) AND TS=((exercise OR sports OR resistance training OR physical activity OR walk OR exercise OR sport OR weight training OR weightlifting OR fitness OR physical activity OR running OR cycling OR swimming)) AND TS=(randomized controlled trial OR random OR placebo OR single blind OR double blind OR triple blind) NOT TS =(comment OR editorial OR meta-analysis OR practice-guideline OR review OR letter OR journal correspondence OR random sample OR random digit OR random effect OR random survey OR random regression) | 2556 |
| **Pubmed** | (Skinfold thickness[Title/Abstract] OR skinfold measurement[Title/Abstract] OR triceps skinfold[Title/Abstract] OR subscapular skinfold[Title/Abstract] OR skinfold caliper[Title/Abstract] OR Waist circumference[Title/Abstract] OR WC[Title/Abstract] OR abdominal circumference[Title/Abstract] OR waist measurement[Title/Abstract] OR obesity[Title/Abstract] OR PWV[Title/Abstract] OR Pulse Wave Velocity[Title/Abstract] OR Arterial stiffness index[Title/Abstract] OR Cardio-Ankle Vascular Index[Title/Abstract] OR ΔCAVI[Title/Abstract] OR Systolic Blood Pressure[Title/Abstract] OR SBP[Title/Abstract] OR Diastolic Blood Pressure[Title/Abstract] OR DBP[Title/Abstract] OR Total Cholesterol[Title/Abstract] OR TC[Title/Abstract] OR Triglycerides[Title/Abstract] OR TG[Title/Abstract] OR High-Density Lipoprotein Cholesterol[Title/Abstract] OR HDL-C[Title/Abstract] OR Low-Density Lipoprotein Cholesterol[Title/Abstract] OR LDL-C[Title/Abstract]) AND(single bout[Title/Abstract] OR multiple bout[Title/Abstract] OR (short[Title/Abstract] AND bout[Title/Abstract]) OR long bout[Title/Abstract] OR one bout[Title/Abstract] OR repeated bout[Title/Abstract] OR continuous[Title/Abstract] OR intermittent[Title/Abstract] OR accumulate*[Title/Abstract] OR break[Title/Abstract] OR breaks[Title/Abstract] OR breaking[Title/Abstract] OR interrupting[Title/Abstract] OR physical activity pattern[Title/Abstract] OR exercise pattern[Title/Abstract] OR intervals[Title/Abstract] OR alternating[Title/Abstract] OR Exercise snack[Title/Abstract] OR periodic exercise[Title/Abstract]) AND (exercise[Title/Abstract] OR sports[Title/Abstract] OR resistance training[Title/Abstract] OR physical activity[Title/Abstract] OR walk[Title/Abstract] OR sport[Title/Abstract] OR weight training[Title/Abstract] OR weightlifting[Title/Abstract] OR fitness[Title/Abstract] OR running[Title/Abstract] OR cycling[Title/Abstract] OR swimming[Title/Abstract]) AND (randomized controlled trial[Title/Abstract] OR random*[Title/Abstract] OR placebo[Title/Abstract] OR single blind[Title/Abstract] OR double blind[Title/Abstract] OR triple blind[Title/Abstract])  NOT (comment[Title/Abstract] OR editorial[Title/Abstract] OR meta-analysis[Title/Abstract] OR practice guideline[Title/Abstract] OR review[Title/Abstract] OR letter[Title/Abstract] OR journal correspondence[Title/Abstract] OR random sample[Title/Abstract] OR random digit[Title/Abstract] OR random effect[Title/Abstract] OR random survey[Title/Abstract] OR random regression[Title/Abstract]) | 1287 |
| **Cochrane library** | #1=(exercise):ti,ab,kw OR (sports):ti,ab,kw OR (resistance training):ti,ab,kw OR (physical activity):ti,ab,kw OR (walk):ti,ab,kw OR (sport):ti,ab,kw OR (weight training):ti,ab,kw OR (weightlifting):ti,ab,kw OR (fitness):ti,ab,kw OR (running):ti,ab,kw OR (cycling):ti,ab,kw OR (swimming):ti,ab,kw  #2=(single bout):ti,ab,kw OR (multiple bout):ti,ab,kw OR (short AND bout):ti,ab,kw OR (long bout):ti,ab,kw OR (one bout):ti,ab,kw OR (repeated bout):ti,ab,kw OR (continuous):ti,ab,kw OR (intermittent):ti,ab,kw OR (accumulate):ti,ab,kw OR (break):ti,ab,kw OR (breaks):ti,ab,kw OR (breaking):ti,ab,kw OR (interrupting):ti,ab,kw OR ("physical activity pattern"):ti,ab,kw OR ("exercise pattern"):ti,ab,kw OR (intervals):ti,ab,kw OR (alternating):ti,ab,kw OR ("exercise snack"):ti,ab,kw OR ("periodic exercise"):ti,ab,kw  #3=("Skinfold thickness"):ti,ab,kw OR ("Skinfold measurement"):ti,ab,kw OR ("triceps skinfold"):ti,ab,kw OR ("subscapular skinfold"):ti,ab,kw OR ("Skinfold caliper"):ti,ab,kw OR ("Waist circumference"):ti,ab,kw OR (WC):ti,ab,kw OR ("abdominal circumference"):ti,ab,kw OR ("waist measurement"):ti,ab,kw OR (obesity):ti,ab,kw OR (PWV):ti,ab,kw OR ("Pulse Wave Velocity"):ti,ab,kw OR ("Arterial stiffness index"):ti,ab,kw OR ("Change in Cardio-Ankle Vascular Index"):ti,ab,kw OR (ΔCAVI):ti,ab,kw OR ("Systolic Blood Pressure"):ti,ab,kw OR (SBP):ti,ab,kw OR ("Diastolic Blood Pressure"):ti,ab,kw OR (DBP):ti,ab,kw OR ("Total Cholesterol"):ti,ab,kw OR (TC):ti,ab,kw OR (Triglycerides):ti,ab,kw OR (TG):ti,ab,kw OR ("High-Density Lipoprotein Cholesterol"):ti,ab,kw OR (HDL-C):ti,ab,kw OR ("Low-Density Lipoprotein Cholesterol"):ti,ab,kw OR (LDL-C):ti,ab,kw  #4=("randomized controlled trial"):ti,ab,kw OR (random*):ti,ab,kw OR (placebo):ti,ab,kw OR ("single blind"):ti,ab,kw OR ("double blind"):ti,ab,kw OR ("triple blind"):ti,ab,kw  #5=(comment):ti,ab,kw OR (editorial):ti,ab,kw OR ("meta-analysis"):ti,ab,kw OR ("practice guideline"):ti,ab,kw OR (review):ti,ab,kw OR (letter):ti,ab,kw OR ("journal correspondence"):ti,ab,kw OR ("random sample"):ti,ab,kw OR ("random digit"):ti,ab,kw OR ("random effect"):ti,ab,kw OR ("random survey"):ti,ab,kw OR ("random regression"):ti,ab,kw #1 AND #2 AND #3 AND #4 NOT # 5 | 1936 |
| **Embase** | (('exercise':ab,ti OR 'sports':ab,ti OR 'resistance training':ab,ti OR 'physical activity':ab,ti OR 'walk':ab,ti OR 'sport':ab,ti OR 'weight training':ab,ti OR 'weightlifting':ab,ti OR 'fitness':ab,ti OR 'running':ab,ti OR 'cycling':ab,ti OR 'swimming':ab,ti) AND ('single bout':ab,ti OR 'multiple bout':ab,ti OR ('short':ab,ti AND 'bout':ab,ti) OR 'long bout':ab,ti OR 'one bout':ab,ti OR 'repeated bout':ab,ti OR 'continuous':ab,ti OR 'intermittent':ab,ti OR 'accumulative':ab,ti OR 'break':ab,ti OR 'breaks':ab,ti OR 'breaking':ab,ti OR 'interrupting':ab,ti OR 'physical activity pattern':ab,ti OR 'exercise pattern':ab,ti OR 'intervals':ab,ti OR 'alternating':ab,ti OR 'exercise snack':ab,ti OR 'periodic exercise':ab,ti) AND ('Skinfold thickness':ab,ti OR 'Skinfold measurement':ab,ti OR 'triceps skinfold':ab,ti OR 'subscapular skinfold':ab,ti OR 'Skinfold caliper':ab,ti OR 'Waist circumference':ab,ti OR 'WC':ab,ti OR 'abdominal circumference':ab,ti OR 'waist measurement':ab,ti OR 'obesity':ab,ti OR 'PWV':ab,ti OR 'Pulse Wave Velocity':ab,ti OR 'Arterial stiffness index':ab,ti OR 'Change in Cardio-Ankle Vascular Index':ab,ti OR 'ΔCAVI':ab,ti OR 'Systolic Blood Pressure':ab,ti OR 'SBP':ab,ti OR 'Diastolic Blood Pressure':ab,ti OR 'DBP':ab,ti OR 'Total Cholesterol':ab,ti OR 'TC':ab,ti OR 'Triglycerides':ab,ti OR 'TG':ab,ti OR 'High-Density Lipoprotein Cholesterol':ab,ti OR 'HDL-C':ab,ti OR 'Low-Density Lipoprotein Cholesterol':ab,ti OR 'LDL-C':ab,ti) AND ('randomized controlled trial':ab,ti OR 'random*':ab,ti OR 'placebo':ab,ti OR 'single blind':ab,ti OR 'double blind':ab,ti OR 'triple blind':ab,ti)) NOT ('comment':ab,ti OR 'editorial':ab,ti OR 'meta-analysis':ab,ti OR 'practice guideline':ab,ti OR 'review':ab,ti OR 'letter':ab,ti OR 'journal correspondence':ab,ti OR 'random sample':ab,ti OR 'random digit':ab,ti OR 'random effect':ab,ti OR 'random survey':ab,ti OR 'random regression':ab,ti) | 2274 |

**Supplementary Table 3 Included Study Characteristics**

| **Author, Year** | **Study Characteristics** | | | | **Training Protocol** | |
| --- | --- | --- | --- | --- | --- | --- |
|  | **Sex** | **Population Characteristics** | **Age (years)** | **Sample Size** | **Intervention**  **(Accumulated / Intermittent)** | **Comparator (Continuous)** |
| Jakicic, 1995 | Female | Obese, sedentary, BMI = 33.9±4.1 kg/m² | 40.4±5.9 (SB group); 40.9±7.3 (LB group) | 56 (2 groups, 28 per group) | Training protocol: 5 days/week, multiple 10-min bouts of exercise per day, total duration progressively increased from 20 min/day to 40 min/day at an intensity of 70% heart rate reserve (HRR); Training interval: No fixed interval between bouts; Training adherence: - | Training protocol: 5 days/week, one continuous bout of exercise per day, total duration progressively increased from 20 min/day to 40 min/day at an intensity of 70% HRR; Training interval: No interval requirement; Training adherence: - |
| Murphy, 1998 | Female | Sedentary, no severe diseases, BMI < 30 kg/m² | 44.4±6.2 | 47 (34 completed, 3 groups) | Training protocol: 5 days/week, 3 bouts of 10-min brisk walking per day at an intensity of 70–80% maximum heart rate (HRmax) for 10 weeks; Training interval: Interval ≥ 4 h between consecutive bouts; Training adherence: 85% ± 2% | Training protocol: 5 days/week, one bout of 30-min brisk walking per day at an intensity of 70–80% HRmax for 10 weeks; Training interval: No interval requirement; Training adherence: 88% ± 2% |
| Coleman, 1999 | Mixed | Sedentary, no severe diseases, normal to slightly overweight BMI | 38.1±7.6 (choice group); 40.6±9.5 (1×30 min group); 41.1±8.9 (3×10 min group) | 32 (3 groups) | Training protocol: 6 days/week, 3 bouts of 10-min brisk walking per day; optional group: 6 days/week, cumulative 30-min brisk walking per day with each bout ≥ 5 min; Training interval: No specified interval between bouts; Training adherence: - | Training protocol: 6 days/week, one bout of 30-min brisk walking per day; Training interval: No interval requirement; Training adherence: - |
| Jakicic, 1999 | Female | Sedentary, overweight, BMI = 32.8±4.0 kg/m² | 36.7±5.6 | 148 (3 groups) | Training protocol: 5 days/week, multiple 10-min bouts of exercise per day, total duration increased from 20 min/day to 40 min/day; SBEQ subgroup: same as SB group plus home treadmill intervention; Training interval: No fixed interval between bouts; Training adherence: Reduction in weekly exercise duration of -68.7 ± 95.1 min during months 13–18; proportion of participants completing ≥ 80% of prescribed exercise: 31.4% | Training protocol: 5 days/week, one continuous bout of exercise per day, total duration increased from 20 min/day to 40 min/day; Training interval: No interval requirement; Training adherence: Reduction in weekly exercise duration of -42.2 ± 73.7 min during months 13–18; proportion of participants completing ≥ 80% of prescribed exercise: 24.5% |
| Schmidt, 2001 | Female | Overweight, BMI ≥ 28 kg/m², college students | 20.8±1.6 (control group); 20.7±2.5 (1×30 group); 18.3±0.48 (2×15 group); 19±0.93 (3×10 group) | 38 (4 groups) | Training protocol: 3–5 days/week, 2 bouts of 15-min exercise per day (2×15 subgroup) / 3 bouts of 10-min exercise per day (3×10 subgroup) at an intensity of 75% HRR for 12 weeks; Training interval: No specified interval between bouts; Training adherence: - | Training protocol: 3–5 days/week, one bout of 30-min exercise per day at an intensity of 75% HRR for 12 weeks; Training interval: No interval requirement; Training adherence: - |
| Murphy, 2002 | Mixed | Sedentary, BMI < 35 kg/m², no severe diseases | 44.5±6.1 | 21 (2 groups, crossover design) | Training protocol: 5 days/week, 3 bouts of 10-min brisk walking per day at an intensity of 70–80% HRmax for 6 weeks; Training interval: 3 h interval between consecutive bouts; Training adherence: 88.2% ± 1.1% | Training protocol: 5 days/week, one bout of 30-min brisk walking per day at an intensity of 70–80% HRmax for 6 weeks; Training interval: No interval requirement; Training adherence: 91.3% ± 4.1% |
| Asikainen, 2003 | Female | Postmenopausal, healthy, sedentary, BMI < 32 kg/m² | 58±4.4 (W1 group); 58±4.2 (W2 group); 57±4.2 (control group) | Study I: 134 (130 completed) | Training protocol: 5 days/week, 2 bouts of exercise per day at an intensity of 65% VO₂max with total energy expenditure of 300 kcal/day; Training interval: No fixed interval between bouts; Training adherence: 88–95% session completion rate; 3 dropouts out of 88 participants | Training protocol: 5 days/week, one bout of exercise per day at an intensity of 65% VO₂max with total energy expenditure of 300 kcal/day; Training interval: No interval requirement; Training adherence: 88–95% session completion rate; 3 dropouts out of 81 participants |
| Murtagh, 2005 | Mixed | Sedentary, BMI < 30 kg/m², no severe diseases | 45.7±9.4 | 32 | Training protocol: 3 days/week, 2 bouts of 10-min brisk walking per day for 12 weeks; Training interval: No fixed interval between bouts; Training adherence: 82.1% ± 10.0% | Training protocol: 3 days/week, one bout of 20-min brisk walking per day for 12 weeks; Training interval: No interval requirement; Training adherence: 90.4% ± 10.6% |
| Altena, 2006 | Mixed | Sedentary, non-obese, normal blood lipid levels | 29.0±2.2 (CON-EX group); 22.0±2.4 (INT-EX group) | 18 (2 groups) | Training protocol: 5 days/week, 3 bouts of 10-min jogging per day at an intensity of 60% VO₂max for 4 weeks; Training interval: 20-min sedentary rest interval between consecutive bouts; Training adherence: 100% | Training protocol: 5 days/week, one bout of 30-min jogging per day at an intensity of 60% VO₂max for 4 weeks; Training interval: No interval requirement; Training adherence: 100% |
| Quinn, 2006 | Mixed | Low to moderate physical activity level, no diseases, no medications for blood pressure or lipid control | 48.8±9.0 | 37 (17 males, 20 females) | Training protocol: 4 days/week, 2 bouts of 15-min exercise per day with intensity progressively increased to 70–80% HRR for 12 weeks; Training interval: ≥ 4 h interval between bouts in the first 12 weeks; crossover to continuous exercise without interval in the subsequent 12 weeks; Training adherence: 96.3% ± 17.7% | Training protocol: 4 days/week, one bout of 30-min exercise per day with intensity progressively increased to 70–80% HRR for 12 weeks; Training interval: No interval in the first 12 weeks; crossover to intermittent exercise with ≥ 4 h interval between bouts in the subsequent 12 weeks; Training adherence: 96.6% ± 12.2% |
| Serwe, 2011 | Female | Inactive (daily exercise < 30 min, ≤ 2 days/week in past 3 months), premenopausal healthcare workers, no cardiovascular diseases, no medications affecting blood pressure, non-pregnant | 36.3–38.5 | 60 (53 completed) | Training protocol: 5 days/week, 3 bouts of 10-min exercise per day at an intensity of 60–70% HRR for 8 weeks; Training interval: No specified interval between bouts; Training adherence: - | Training protocol: 5 days/week, one bout of 30-min exercise per day at an intensity of 60–70% HRR for 8 weeks; a blank control group (no exercise) was additionally set; Training interval: No interval requirement; Training adherence: - |
| Alizadeh, 2013 | Female | Overweight or obese (BMI ≥ 25), no regular exercise history, no smoking or significant weight change in past 6 months, no diseases affecting body weight, no medications affecting heart rate or appetite | 32.4–34.5 | 45 (31 completed) | Training protocol: 5 days/week, 3 bouts of exercise per day (each ≥ 10 min) with total duration of 40 min/day at an intensity of 64–76% HRmax for 12 weeks; Training interval: Interval > 4 h between consecutive bouts; Training adherence: 5 dropouts out of 15 participants | Training protocol: 5 days/week, one bout of 40-min exercise per day at an intensity of 64–76% HRmax for 12 weeks; a blank control group (no exercise) was additionally set; Training interval: No interval requirement; Training adherence: 6 dropouts out of 15 participants |
| Eguchi, 2013 | Male | Sedentary (daily exercise < 20 min, < 3 days/week in past 6 months), with cardiovascular risk factors (e.g., obesity, mild hypertension), no cardiovascular diseases or musculoskeletal problems | 43.9±11.6 | 23 | Training protocol: 3 days/week, 3 bouts of 10-min exercise per day at an intensity of 50% maximal oxygen uptake (VO₂max) for 20 weeks; Training interval: No specified interval between bouts; Training adherence: - | Training protocol: 3 days/week, one bout of 30-min exercise per day at an intensity of 50% VO₂max for 20 weeks; Training interval: No interval requirement; Training adherence: - |
| Manthou, 2015 | Female | Overweight or obese, sedentary (planned exercise < 1 hour/week), premenopausal, non-smoking, no medications affecting lipid or carbohydrate metabolism, no special diet, non-pregnant, no hypertension | 31.3–32.1 | 34 | Training protocol: 5 days/week, one bout of 30-min exercise per day at an intensity based on individual lactate threshold for 8 weeks; Training interval: No interval (completed in a single bout); Training adherence: - | Training protocol: 2 days/week, one bout of 75-min exercise per day at an intensity based on individual lactate threshold for 8 weeks; Training interval: No interval requirement (completed in a single bout); Training adherence: - |
| Reynolds, 2016 | Mixed | Type 2 diabetes mellitus patients, aged 18–75, non-pregnant or lactating, able to comply with exercise protocol | 60±9.9 | 42 (41 completed) | Training protocol: 3 bouts of 10-min exercise per day (postprandial) with total duration of 30 min/day for 2 weeks; Training interval: Postprandial interval (no specified duration); Training adherence: - | Training protocol: One bout of 30-min exercise per day (at any time) for 2 weeks; Training interval: No interval requirement; Training adherence: - |
| Chung, 2017 | Female | Middle-aged obese females, body fat percentage ≥ 30%, no regular exercise in past 6 months, no exercise contraindications (e.g., unstable cardiovascular diseases) | 47.75–49.55 | 47 (36 completed) | Training protocol: 3 days/week, 3 bouts of 10-min exercise per day at an intensity of 83.12% ± 3.93% VO₂max for 12 weeks; a blank control group (no exercise) was additionally set; Training interval: No specified interval between bouts; Training adherence: - | Training protocol: 3 days/week, one bout of 30-min exercise per day at an intensity of 83.12% ± 3.93% VO₂max for 12 weeks; a blank control group (no exercise) was additionally set; Training interval: No interval requirement; Training adherence: - |
| Madjd, 2019 | Female | Overweight or obese (BMI 27–35 kg/m²), sedentary (exercise < 3 days/week, < 20 min/day in past 6 months), no cardiovascular diseases, non-smoking, non-pregnant | 27.6–27.9 | 65 (57 completed) | Training protocol: 6 days/week, 2 bouts of 25-min exercise per day at moderate intensity (≥ 100 steps/min) for 24 weeks; Training interval: No specified interval between bouts; Training adherence: - | Training protocol: 6 days/week, one bout of 50-min exercise per day at moderate intensity (≥ 100 steps/min) for 24 weeks; Training interval: No interval requirement; Training adherence: - |
| Murphy, 2000 | Mixed | Sedentary adults; BMI 24–35  kg/m²; 7 postmenopausal females, 3 males; normal blood lipid levels except 1 male with borderline hyperlipidemia | 34–66 | 10 | Training protocol: 5 days/week, 3 bouts of 10-min brisk walking per day at an intensity of 70–80% maximum heart rate (HRmax) for 10 weeks; Training interval: ≥ 4 h between consecutive bouts; Training adherence: 85% ± 2% of the prescribed total exercise duration completed (1298 ± 114 min vs. 1500 min prescribed) | Training protocol: 5 days/week, 1 bout of 30-min brisk walking per day at an intensity of 70–80% HRmax for 10 weeks; Training interval: No interval requirement; Training adherence: 88% ± 2% of the prescribed total exercise duration completed (1316 ± 111 min vs. 1500 min prescribed) |
| Altena, 2004 | Mixed | Inactive; normolipidemic; BMI 23.2±0.8 kg/m²; VO₂max 38.4±1.5 mL·kg⁻¹·min⁻¹ | 25±1.8 | 18 (7 males, 11 females) | Training protocol: 5 days/week, multiple 10-min bouts of exercise per day with total duration progressively increased from 20 min/day to 40 min/day; Training interval: No fixed interval, completed flexibly during daytime; Training adherence: Weekly exercise duration reduction of -68.7 ± 95.1 min during months 13–18; 31.4% of participants completed ≥ 80% of the prescribed exercise | Training protocol: 5 days/week, 1 continuous bout of exercise per day with total duration progressively increased from 20 min/day to 40 min/day; Training interval: No interval requirement; Training adherence: Weekly exercise duration reduction of -42.2 ± 73.7 min during months 13–18; 24.5% of participants completed ≥ 80% of the prescribed exercise |
| Park, 2006 | Mixed | Prehypertensive; BMI 27.0±1.2 kg/m²; VO₂peak 34.5±1.64 mL/kg·min | 47.2±2.92 | 20 (15 males, 5 females) | Training protocol: 10-min brisk walking before each meal, 3 bouts per day; Training interval: Scheduled according to meal times; Training adherence: - | Training protocol: 30-min brisk walking before breakfast per day; Training interval: No interval requirement; Training adherence: - |
| Miyashita, 2008 | Male | Healthy young men; BMI < 35 kg/m²; body fat percentage 11.2±0.9%; VO₂max 56.3±2.1 mL·kg⁻¹·min⁻¹ | 23.4±0.8 | 15 | Training protocol: 5 days/week, 3 bouts of 10-min brisk walking per day at an intensity of 70–80% HRmax for 6 weeks; Training interval: 3 h between consecutive bouts; Training adherence: 88.2% ± 1.1% of the prescribed total exercise duration completed | Training protocol: 5 days/week, 1 bout of 30-min brisk walking per day at an intensity of 70–80% HRmax for 6 weeks; Training interval: No interval requirement; Training adherence: 91.3% ± 4.1% of the prescribed total exercise duration completed |
| Miyashita, 2008 | Male | Obese; BMI 25–37 kg/m²; sedentary; waist circumference 89.2±3.1 cm; systolic blood pressure 131±4 mmHg, diastolic blood pressure 82±5 mmHg | 26.5±1.5 | 8 | Training protocol: 5 days/week, 2 bouts of exercise per day with total energy expenditure of 300 kcal/day at an intensity of 65% maximal oxygen uptake (VO₂max); Training interval: No specified fixed interval; Training adherence: 88–95% of the prescribed exercise sessions completed; 3 dropouts out of 88 participants | Training protocol: 5 days/week, 1 bout of exercise per day with total energy expenditure of 300 kcal/day at an intensity of 65% VO₂max; Training interval: No interval requirement; Training adherence: 88–95% of the prescribed exercise sessions completed; 3 dropouts out of 81 participants |
| Miyashita, 2011 | Male | Normotensive/pre-hypertensive; recreationally active; BMI < 35 kg/m² | 25.0±4.2 | 10 | Training protocol: 3 bouts of 10-min treadmill running per day at an intensity of 60% VO₂max, total duration of 30 min/day; Training interval: 20-min rest between consecutive bouts; Training adherence: - | Training protocol: 1 continuous bout of 30-min treadmill running per day at an intensity of 60% VO₂max; Training interval: No interval requirement; Training adherence: - |
| Peddie, 2013 | Mixed | Healthy, normal-weight; sedentary occupation; weekly exercise < 2.5 hours; fasting blood glucose < 6.1 mmol/L; blood pressure < 140/90 mmHg | 25.9±5.3 | 70 | Training protocol: 3 days/week, 2 bouts of 10-min brisk walking per day for 12 weeks; Training interval: No specified fixed interval; Training adherence: 82.1% ± 10.0% of the prescribed exercise sessions completed (range: 67.6%–100%) | Training protocol: 3 days/week, 1 bout of 20-min brisk walking per day for 12 weeks; Training interval: No interval requirement; Training adherence: 90.4% ± 10.6% of the prescribed exercise sessions completed (range: 76.4%–97.3%) |
| Kim, 2014 | Male | Healthy, non-smoking, normal blood pressure, BMI < 30 kg/m², no history of cardiopulmonary or metabolic diseases, no medications affecting lipid or carbohydrate metabolism | 24.0±4.0 | 9 | Training protocol: 5 days/week, 3 bouts of 10-min jogging per day at an intensity of 60% VO₂max for 4 weeks; Training interval: 20-min sedentary rest between consecutive bouts; Training adherence: - | Training protocol: 5 days/week, 1 bout of 30-min jogging per day at an intensity of 60% VO₂max for 4 weeks; Training interval: No interval requirement; Training adherence: - |
| Zheng, 2015 | Male | Healthy, non-smoking, normal blood pressure, no systematic training history or discontinued systematic training for at least 1 year, no medications for diabetes, metabolic or cardiovascular diseases | 22.0±0.3 | 16 | Training protocol: 4 bouts of 10-min walking per day at an intensity of 50% peak oxygen uptake (VO₂peak); Training interval: Once per hour, 60-min interval between bouts, total duration of 4 h; Training adherence: - | Training protocol: 1 bout of 40-min walking per day at an intensity of 50% VO₂peak; a non-exercise blank control group was additionally set; Training interval: No interval requirement; Training adherence: - |
| Zhou, 2015 | Male | Healthy, no cardiovascular, respiratory or endocrine diseases (including diabetes), non-smoking, no prescription drugs, no participation in university competitive sports | 24.7±0.3 | 19 | Training protocol: 4 days/week, 2 bouts of 15-min exercise per day with intensity progressively increased to 70–80% heart rate reserve (HRR) for 24 weeks; Training interval: ≥ 4 h between bouts in the first 12 weeks, crossover to continuous exercise without interval in the subsequent 12 weeks; Training adherence: 96.3% ± 17.7% of the prescribed exercise duration completed; 70.1% ± 26.1% of the exercise time spent within the target heart rate zone | Training protocol: 4 days/week, 1 bout of 30-min exercise per day with intensity progressively increased to 70–80% HRR for 24 weeks; Training interval: No interval in the first 12 weeks, crossover to intermittent exercise with ≥ 4 h between bouts in the subsequent 12 weeks; Training adherence: 96.6% ± 12.2% of the prescribed exercise duration completed; 69.1% ± 25.5% of the exercise time spent within the target heart rate zone |
| Miyashita, 2016 | Female | Postmenopausal, non-smoking, no history of cardiovascular diseases, BMI < 30 kg/m², blood pressure < 140/90 mmHg, no medications affecting lipid or cardiovascular metabolism, non-compliant with physical activity guidelines | 68.8±3.2 | 15 | Training protocol: 18 bouts of 1-min 40-s brisk walking per day, total duration of 30 min/day at an intensity of 60% maximal aerobic capacity; Training interval: Once every 30 min; Training adherence: - | Training protocol: 1 continuous bout of 30-min brisk walking per day at an intensity of 60% maximal aerobic capacity; Training interval: No interval requirement; Training adherence: - |
| Bhammar, 2017 | Mixed | Overweight/obese (BMI ≥ 25  kg/m²), non-smoking, inactive, no hypertension (blood pressure < 140/90 mmHg), no medications for hypercholesterolemia, diabetes, hypertension or heart diseases | 32±5 | 10 | Training protocol: 9 bouts of intermittent walking per day (once per hour from 09:00 to 18:00), 30 min for the first bout, 60 min for the last bout, and an average of 17.8 ± 4.0 min per bout for the middle 7 bouts; Training interval: 60-min interval between consecutive bouts; Training adherence: - | Training protocol: 1 bout of 60-min running per day; Training interval: No interval requirement; Training adherence: - |
| Engeroff, 2017 | Female | Healthy, premenopausal, blood pressure < 140/90 mmHg, BMI < 30 kg/m², no medications affecting metabolism, non-smoking | 25.6±2.6 | 18 | Training protocol: 5 days/week, 3 bouts of exercise per day (each > 10 min) with total duration of 40 min/day at an intensity of 64–76% HRmax for 12 weeks; Training interval: > 4 h between consecutive bouts; Training adherence: - | Training protocol: 5 days/week, 1 bout of 40-min exercise per day at an intensity of 64–76% HRmax for 12 weeks; Training interval: No interval requirement; Training adherence: - |
| Fonseca, 2018 | Male | Prehypertensive, recreationally active (2–5 sessions/week, 20–60 min/session for at least 6 months), no cardiovascular diseases or diabetes, no medications affecting cardiovascular response, non-smoking, no musculoskeletal problems | 23–34 | 10 | Training protocol: 2 bouts of 15-min cycling per day at an intensity of 50% HRR; Training interval: 20-min interval for AE20 subgroup, 60-min interval for AE60 subgroup; Training adherence: - | Training protocol: 1 bout of 30-min cycling per day at an intensity of 50% HRR; Training interval: No interval requirement; Training adherence: - |
| Kashiwabara, 2018 | Female | Postmenopausal, hypertriglyceridemia (fasting TG ≥ 1.70 mmol/L), non-smoking, no major diseases, no physical limitations to low-intensity exercise, non-compliant with physical activity guidelines | 70.5±4.6 | 12 | Training protocol: 3 bouts of 10-min cycling per day at an intensity of 50% HRR for both AE10 and AE60 subgroups; Training interval: 10-min interval for AE10 subgroup, 60-min interval for AE60 subgroup; Training adherence: - | Training protocol: 1 continuous bout of 30-min cycling per day at an intensity of 50% HRR; Training interval: No interval requirement; Training adherence: - |
| Shambrook, 2020 | Mixed | Insufficiently physically active, no diagnosed cardiometabolic diseases, no related medications | 50±12.6 | 10 | Training protocol: 20 bouts of 1.5-min brisk walking per day, total duration of 30 min/day at self-selected speed; Training interval: No fixed interval, distributed flexibly throughout the day; Training adherence: - | Training protocol: 1 continuous bout of 30-min brisk walking in the morning per day at self-selected speed; Training interval: No interval requirement; Training adherence: - |
| Zhou, 2022 | Male | Healthy young males, non-smoking, no cardiovascular system-related diseases | 22.4±0.4 | 20 | Training protocol: 21 bouts of 2-min moderate-intensity walking per day for 2min-MOD subgroup, 8 bouts of 2-min vigorous-intensity walking per day for 2min-VIG subgroup; Training interval: Once every 20 min for 2min-MOD subgroup, once every hour for 2min-VIG subgroup; Training adherence: - | Training protocol: 1 continuous bout of 30-min walking per day at an intensity of 71 ± 4% HRmax; Training interval: No interval requirement; Training adherence: - |
| Liu, 2024 | Male | Healthy young males, no regular physical exercise, no obesity, heart diseases, etc. | 18.76±1.81 | 30 | Training protocol: 5 bouts of 6-min cycling per day, total duration of 30 min/day at an intensity of 70% VO₂max; Training interval: Distributed flexibly within 4 h; Training adherence: - | Training protocol: 1 continuous bout of 30-min cycling before sedentary behavior per day at an intensity of 70% VO₂max; Training interval: No interval requirement; Training adherence: - |

Note: HRR = Heart Rate Reserve, HRmax = Maximal Heart Rate, VO₂max = Maximal Oxygen Uptake, VO₂peak = Peak Oxygen Uptake, HIT = High-Intensity Interval Training, MCT = Moderate-Intensity Continuous Training, TG = Triglyceride, CST = Constant-Load Training, VO₂R = Oxygen Uptake Reserve, SB = Short Bout, LB = Long Bout

Supplementary Table 4 Methodological quality assessment ( PEDro )

| Author , year | D1 | D2 | D3 | D4 | D5 | D6 | D7 | D8 | D9 | D10 | D11 | Total |
| --- | --- | --- | --- | --- | --- | --- | --- | --- | --- | --- | --- | --- |
| Jakicic,1995 | Y | 0 | 1 | 0 | 0 | 1 | 1 | 1 | 1 | 1 | 1 | 7 |
| Murphy,1998 | Y | 0 | 1 | 0 | 0 | 1 | 0 | 1 | 1 | 1 | 1 | 6 |
| Coleman,1999 | Y | 0 | 1 | 0 | 0 | 1 | 1 | 1 | 1 | 1 | 1 | 7 |
| Jakicic,1999 | Y | 0 | 1 | 0 | 0 | 1 | 1 | 1 | 1 | 1 | 1 | 7 |
| Murphy,2002 | Y | 0 | 1 | 0 | 0 | 1 | 0 | 1 | 1 | 1 | 1 | 6 |
| Asikainen,2003 | Y | 0 | 1 | 0 | 0 | 1 | 1 | 1 | 1 | 1 | 1 | 7 |
| Murtagh,2005 | Y | 0 | 1 | 0 | 0 | 1 | 0 | 1 | 1 | 1 | 0 | 5 |
| Quinn,2006 | Y | 1 | 1 | 0 | 0 | 0 | 0 | 1 | 1 | 1 | 1 | 6 |
| Serwe,2011 | Y | 0 | 1 | 0 | 0 | 0 | 1 | 1 | 1 | 1 | 1 | 6 |
| Alizadeh,2013 | Y | 1 | 1 | 0 | 0 | 0 | 0 | 1 | 1 | 1 | 1 | 6 |
| Eguchi,2013 | Y | 1 | 1 | 0 | 0 | 0 | 0 | 1 | 1 | 1 | 1 | 6 |
| Manthou,2015 | Y | 1 | 1 | 0 | 0 | 0 | 0 | 1 | 1 | 1 | 1 | 6 |
| Reynolds,2016 | Y | 1 | 1 | 0 | 0 | 0 | 1 | 1 | 1 | 1 | 1 | 7 |
| Chung,2017 | Y | 1 | 1 | 0 | 0 | 0 | 0 | 1 | 1 | 1 | 1 | 6 |
| Madjd,2019 | Y | 1 | 1 | 0 | 0 | 0 | 1 | 1 | 1 | 1 | 1 | 7 |
| Murphy,2000 | Y | 0 | 1 | 0 | 0 | 0 | 0 | 0 | 1 | 1 | 1 | 4 |
| Park,2006 | Y | 0 | 1 | 0 | 0 | 0 | 0 | 1 | 1 | 1 | 1 | 5 |
| Miyashita,2008 | Y | 0 | 1 | 0 | 0 | 0 | 0 | 0 | 1 | 1 | 1 | 4 |
| Miyashita,2008 | Y | 0 | 1 | 0 | 0 | 0 | 0 | 0 | 1 | 1 | 1 | 4 |
| Miyashita,2011 | Y | 0 | 1 | 0 | 0 | 0 | 0 | 1 | 1 | 1 | 1 | 5 |
| Peddie,2013 | Y | 0 | 1 | 0 | 0 | 0 | 1 | 1 | 1 | 1 | 1 | 6 |
| Kim,2014 | Y | 0 | 1 | 0 | 0 | 0 | 0 | 0 | 1 | 1 | 1 | 4 |
| Zheng,2015 | Y | 0 | 1 | 0 | 0 | 0 | 0 | 0 | 1 | 1 | 1 | 4 |
| Zhou,2015 | Y | 0 | 1 | 0 | 0 | 0 | 0 | 0 | 1 | 1 | 1 | 4 |
| Miyashita,2016 | Y | 0 | 1 | 0 | 0 | 0 | 0 | 0 | 1 | 1 | 1 | 4 |
| Bhammar,2017 | Y | 0 | 1 | 0 | 0 | 0 | 0 | 1 | 1 | 1 | 1 | 5 |
| Engeroff,2017 | Y | 0 | 1 | 0 | 0 | 0 | 0 | 0 | 1 | 1 | 1 | 4 |
| Fonseca,2018 | Y | 0 | 1 | 0 | 0 | 0 | 0 | 0 | 1 | 1 | 1 | 4 |
| Kashiwabara,2018 | Y | 0 | 1 | 0 | 0 | 0 | 0 | 0 | 1 | 1 | 1 | 4 |
| Shambrook,2020 | Y | 1 | 1 | 0 | 0 | 0 | 0 | 0 | 1 | 1 | 1 | 5 |
| Zhou,2022 | Y | 1 | 1 | 0 | 0 | 0 | 0 | 0 | 1 | 1 | 1 | 5 |
| Liu,2024 | Y | 1 | 1 | 0 | 0 | 0 | 0 | 0 | 1 | 1 | 1 | 5 |

Note: 1. Random allocation (used solely to determine if the study is an RCT and not for scoring); 2. Concealment of the random allocation scheme; 3. Baseline comparability of participants; 4. Blinding of participants; 5. Blinding of therapists; 6. Blinding of outcome assessors; 7. Intention-to-treat analysis; 8. Description of withdrawals or dropouts; 9. Measurement of at least one key outcome indicator; 10. Reliability of key outcome indicator measurement; 11. Statistical significance or effect size of between-group differences in key outcome indicators. The scoring range is 0 to 10 points, where ≥6 points indicate high quality, 4 to 5 points indicate moderate quality, and ≤3 points indicate low quality.

Supplementary Table 5 Methodological quality assessment ( MINORS )

| Author, year | D1 | D2 | D3 | D4 | D5 | D6 | D7 | D8 | Total |
| --- | --- | --- | --- | --- | --- | --- | --- | --- | --- |
| Schmidt,2001 | 2 | 2 | 1 | 2 | 0 | 2 | 2 | 2 | 15 |
| Altena,2006 | 2 | 2 | 1 | 2 | 1 | 2 | 2 | 2 | 16 |
| Altena,2004 | 2 | 2 | 1 | 2 | 1 | 2 | 2 | 2 | 16 |

Note: D1=Clear statement of the study purpose; D2=Clear inclusion and exclusion criteria; D3=Appropriate recruitment of study participants; D4=Appropriate choice of control group; D5=Baseline comparability of groups; D6=Clear description of the intervention; D7=Assessment of at least one key outcome; D8=Adequate follow-up period and outcome assessment Each item was scored as 0 (not reported), 1 (partially reported), or 2 (fully reported). Randomized controlled trials (RCTs) were scored from D1 to D12 with a total score of 0–24, while non-randomized controlled trials (NRCTs) were scored from D1 to D8 only with a total score of 0–16.

|   Supplementary Figure 1 A Risk of bias for the included studies (RoB 2) | SuppleSupplementary Figure 1 B Risk of bias for the included studies (ROBINS‑i) |
| --- | --- |

|   Supplementary Figure 2 A. Sensitivity analysis plot of BF |   Supplementary Figure 2 B. Sensitivity analysis plot of LBM |   Supplementary Figure 2 C. Sensitivity analysis plot of BW |
| --- | --- | --- |
|   Supplementary Figure 2 D. Sensitivity analysis plot of WC |   Supplementary Figure 2 E.Sensitivity analysis plot of SBP |   Supplementary Figure 2 F.Sensitivity analysis plot of DBP |
|   Supplementary Figure 2 G.Sensitivity analysis plot of TC |   Supplementary Figure 2 H.Sensitivity analysis plot of TG |   Supplementary Figure 2 I.Sensitivity analysis plot of HDL - C |
|   Supplementary Figure 2 J.Sensitivity analysis plot of LDL - C |   Supplementary Figure 2 K.Sensitivity analysis plot of S-FTG |   Supplementary Figure 2 L.Sensitivity analysis plot of S-TG-iAUC |
|   Supplementary Figure 2 M.Sensitivity analysis plot of S-SBP |   Supplementary Figure 2 N.Sensitivity analysis plot of S-DBP |   Supplementary Figure 2 O.Sensitivity analysis plot of S-ASI |

Note: BF = Body Fat, LBM= Lean Body Mass,BW = Body Weight, WC = Waist Circumference, SBP = Systolic Blood Pressure, DBP = Diastolic Blood Pressure, TC = Total Cholesterol, TG = Triglyceride, HDL-C = High-Density Lipoprotein Cholesterol, LDL-C = Low-Density Lipoprotein Cholesterol, S-FTG = Single-exercise Fasting Triglyceride, S-TG-iAUC = Single-exercise Incremental Area Under the Curve of Triglyceride, S-SBP = Single-exercise Systolic Blood Pressure, S-DBP = Single-exercise Diastolic Blood Pressure,S-ASI = Single-exercise Arterial Stiffness-Associated Markers.

|   Supplementary Figure 3 A. Forest plot of BF |   Supplementary Figure 3 B. Forest plot of LBM |   Supplementary Figure 3 C. Forest plot of BW |
| --- | --- | --- |
|   Supplementary Figure 3 D. Forest plot of WC |   Supplementary Figure 3 E.Forest plot of SBP |   Supplementary Figure 3 F.Forest plot of DBP |
|   Supplementary Figure 3 G.Forest plot of TC |   Supplementary Figure 3 H.Forest plot of TG |   Supplementary Figure 3 I.Forest plot of HDL - C |
|   Supplementary Figure 3 J.Forest plot of LDL - C |   Supplementary Figure 3 K.Forest plot of S-FTG |   Supplementary Figure 3 L.Forest plot of S-TG-iAUC |
|   Supplementary Figure 3 M.Forest plot of S-SBP |   Supplementary Figure 3 N.Forest plot of S-DBP |   Supplementary Figure 3 O.Forest plot of S-ASI |

Note: BF = Body Fat, LBM= Lean Body Mass,BW = Body Weight, WC = Waist Circumference, SBP = Systolic Blood Pressure, DBP = Diastolic Blood Pressure, TC = Total Cholesterol, TG = Triglyceride, HDL-C = High-Density Lipoprotein Cholesterol, LDL-C = Low-Density Lipoprotein Cholesterol, S-FTG = Single-exercise Fasting Triglyceride, S-TG-iAUC = Single-exercise Incremental Area Under the Curve of Triglyceride, S-SBP = Single-exercise Systolic Blood Pressure, S-DBP = Single-exercise Diastolic Blood Pressure,S-ASI = Single-exercise Arterial Stiffness-Associated Markers.

Supplementary Table 6 Sensitivity analyses of pooled Hedges’ g across three intra-subject correlation coefficients (r = 0.2, 0.5, 0.8)

| Outcome/Subgroup | | r = 0.2 | Primary analysis r = 0.5 | r = 0.8 | Consistency of conclusion |
| --- | --- | --- | --- | --- | --- |
|  |  | Hedges' g (95% CI) | Hedges' g (95% CI) | Hedges' g (95% CI) |  |
| BF | | -0.01 (-0.18, 0.17) | -0.02 (-0.26, 0.22) | -0.02 (-0.33, 0.29) | Non-significant across all r |
| LBM | | 0.02 (-0.15, 0.19) | 0.03 (-0.22, 0.27) | 0.04 (-0.14, 0.21) | Non-significant across all r |
| BW | | -0.18 (-0.37, 0.00)^[1]^ | -0.20 (-0.39, -0.01) | -0.23 (-0.45, -0.01) | Significant only at r=0.5 / r=0.8; non-significant at r=0.2^1^ |
| WC | | 0.01 (-0.11, 0.12) | 0.01 (-0.16, 0.17) | 0.01 (-0.12, 0.13) | Non-significant across all r |
| SBP | | -0.12 (-0.12, 0.37) | -0.18 (-0.17, 0.52) | -0.25 (-0.14, 0.64) | Non-significant across all r |
| DBP | | 0.10 (-0.08, 0.29) | 0.16 (-0.10, 0.42) | 0.20 (-0.11, 0.50) | Non-significant across all r |
| TC | | -0.02 (-0.22, 0.16) | -0.03 (-0.30, 0.23) | -0.05 (-0.32, 0.24) | Non-significant across all r |
| TG | | -0.02 (-0.20, 0.15) | -0.03 (-0.28, 0.22) | -0.05 (-0.22, 0.13) | Non-significant across all r |
| HDL-C | | 0.07 (-0.11, 0.25) | 0.09 (-0.16, 0.35) | 0.12 (-0.18, 0.47) | Non-significant across all r |
| LDL-C | | -0.08 (-0.30, 0.15) | -0.10 (-0.41, 0.22) | -0.15(-0.48, 0.32) | Non-significant across all r |
| S-FTG | - | -0.06 (-0.49, 0.37) | -0.08 (-0.44, 0.28) | -0.11 (-0.39, 0.17) | Non-significant across all r |
| S-TG-iAUC | - | 0.19 0.01 0.44) | 0.24 (0.02, 0.46) | 0.29 (0.09, 0.49) | All statistically significant |
| S-SBP | 10–20 min | -0.51 (-0.98, -0.04) | -0.61 (-1.03, -0.18) | -0.72 (-1.09, -0.35) | All statistically significant |
|  | 20–40 min | -0.39 (-0.94, 0.16) | -0.47 (-0.97, 0.03) | -0.56 (-0.99, -0.12) | Non-significant across all r |
|  | 40–60 min | -0.08 (-0.53, 0.37) | -0.10 (-0.50, 0.30) | -0.12 (-0.47, 0.23) | Non-significant across all r |
|  | 24 h | -0.13 (-0.88, 0.62) | -0.16 (-0.84, 0.52) | -0.19 (-0.80, 0.42) | Non-significant across all r |
| S-DBP | 10–20 min | -0.12 (-0.47, 0.23) | -0.14 (-0.43, 0.15) | -0.17 (-0.39, 0.05) | Non-significant across all r |
|  | 20–40 min | -0.53 (-0.95, -0.11) | -0.61 (-1.11, -0.10) | -0.79 (-1.45, -0.12) | All statistically significant |
|  | 40–60 min | 0.02 (-0.42, 0.46) | 0.02 (-0.38, 0.42) | 0.03 (-0.34, 0.40) | Non-significant across all r |
|  | 24 h | 0.08 (-0.43, 0.59) | 0.10 (-0.37, 0.57) | 0.12 (-0.30, 0.54) | Non-significant across all r |
| S-ASI | PWV | -0.63 (-1.62, 0.06) | -0.76 (-1.67, 0.16) | -0.90 (-1.72, 0.02) | Non-significant across all r |
|  | ASIβ | 0.16 (-0.30, 0.62) | 0.19 (-0.26, 0.63) | 0.23 (-0.21, 0.67) | Non-significant across all r |
|  | ⊿CAVI | -0.47 (-0.92, -0.02) | -0.56 (-0.96, -0.16) | -0.66 (-1.00, -0.32) | All statistically significant |

Note: BF = Body Fat, LBM= Lean Body Mass,BW = Body Weight, WC = Waist Circumference, SBP = Systolic Blood Pressure, DBP = Diastolic Blood Pressure, TC = Total Cholesterol, TG = Triglyceride, HDL-C = High-Density Lipoprotein Cholesterol, LDL-C = Low-Density Lipoprotein Cholesterol, S-FTG = Single-exercise Fasting Triglyceride, S-TG-iAUC = Single-exercise Incremental Area Under the Curve of Triglyceride, S-SBP = Single-exercise Systolic Blood Pressure, S-DBP = Single-exercise Diastolic Blood Pressure,S-ASI = Single-exercise Arterial Stiffness-Associated Markers.

1. The 95% CI for BW derived from Stata computation was (0.368, 0.000). Considering that the upper bound may represent an infinitesimally small positive value, the pooled effect was deemed statistically non-significant under the r=0.2 scenario.
